# The effect of combined knockdowns of Attacins on survival and bacterial load in *Tenebrio molitor*

**DOI:** 10.3389/fimmu.2023.1140627

**Published:** 2023-03-28

**Authors:** Maryam Keshavarz, Caroline Zanchi, Jens Rolff

**Affiliations:** Evolutionary Biology, Institute of Biology, Freie Universität Berlin, Berlin, Germany

**Keywords:** RNA interference, antimicrobial peptides (AMPs), host survival, Attacin, *Pseudomonas entomophila*, *Tenebrio molitor*

## Abstract

**Introduction:**

Upon infection, insect hosts simultaneously express a cocktail of antimicrobial peptides (AMPs) which can impede pathogen colonization and increase host fitness. It has been proposed that such a cocktail might be adaptive if the effects of co-expressed AMPs are greater than the sum of individual activities. This could potentially prevent the evolution of bacterial resistance. However, *in vivo* studies on AMPs in combination are scarce. Attacins are one of the relatively large AMP families, which show anti-Gram-negative activity *in vitro*.

**Material and methods:**

Here, we used RNA interference (RNAi) to silence three members of the Attacin family genes in the mealworm beetle, *Tenebrio molitor*: (*TmAttacin1a* (*TmAtt1a*), *TmAttacin1b* (*TmAtt1b*), and *TmAttacin*2 (*TmAtt2*) both individually and in combination. We then infected *T. molitor* with the Gram negative entomopathogen *Pseudomonas entomophila*.

**Results:**

We found that survival of the beetles was only affected by the knockdown of *TmAttacin1b*, *TmAttacin2* and the knockdown of all three Attacins together. Triple knockdown, rather than individual or double knockdowns of AMPs, changes the temporal dynamics of their efficiency in controlling the colonization of *P. entomophila* in the insect body.

**Discussion:**

More precisely, AMP gene expression influences *P. entomophila* load early in the infection process, resulting in differences in host survival. Our results highlight the importance of studying AMP-interactions *in vivo*.

## 1 Introduction

Hosts ranging from insects to mammals use conserved innate immune pathways for effective protection from pathogens. The downstream effectors of NF-κB signaling pathways, the antimicrobial peptides (AMPs) or host defense peptides, are inducible short proteins that play versatile roles in host physiology such as antimicrobial and anti-tumor activities and wound healing (1–4). Prior *in vitro* studies have found that AMP expression is long-lasting in several insect species and, remarkably, the expressed cocktails contain some AMPs that are not effective against the infective agent in isolation (5–8). Insects have been reported to simultaneously synthesize an effective repertoire of AMP combinations, either AMP members of the same family or different families (9–11). In addition, AMPs exhibit a greater effect in combination with other AMPs, which suggests that the immune system produces synergistic combinations to enhance their activities upon infections (3, 12).

Until recently, little was known about the *in vivo* AMP activities of single AMPs or AMPs in combination. Zanchi et al. (3) reported the importance of AMP cocktails in determining survival in persistent experimental *Staphylococcus aureus* infections, using RNAi-based single and double knockdown of three highly induced *Tenebrio molitor* AMPs (*Tenecin 1*, *Tenecin 2*, and *Tenecin 4*). In another study using CRISPR/Cas9-Induced loss-of-function, Hanson et al. (13) knocked out 10 out of 14 AMPs individually and in selected combinations and generated three group of flies including Defensin mutants, Drosocin/Attacins/Diptericins mutants, and Metchnikowin/Drosomycin mutants. They found that there are either additive or synergistic combinations of AMPs with either anti-Gram-negative or anti-fungal activities in *Drosophila melanogaster*, as well as *in vivo* specific AMP-pathogen interactions (3, 13). Moreover, insects express several AMPs of the same family simultaneously, including within the Attacin gene family.

After the first identification of Attacins in *Hyalophora cecropia* in 1983 (14), different Attacins from other insect orders including Diptera (*D. melanogaster*, *Glossina morsitans*, and *Hermetia illucens*) (15–18), Lepidoptera (*Hyphantria cunea, Spodoptera exigua*) (19–21), and Coleoptera (*T. molitor*) (22) have been described, and their antimicrobial activities have been investigated. The widespread presence of Attacin peptides in numerous insect orders suggest that Attacins are evolutionarily conserved (23, 24). Attacins as relatively large proteins have antimicrobial activities against Gram-negative bacteria (e.g., *Escherichia coli*) (15, 16, 19, 22). Moreover, earlier studies underlined potential pharmacological applications of Attacins. For example, *in vitro* inhibitory activity of the parasite, *Trypanosoma brucei* by the purified recombinant Attacin from *G. m. morsitans* (15), and also antibacterial activity against methicillin-resistant *S. aureus* (MRSA) by a recombinant Attacin from *H. illucens* (17). However, these studies either characterized the function of the Attacins *in vivo* or investigated the *in vitro* antimicrobial activity individually. The extent, however, to which *in vitro* activities of AMPs reflect the effects of their expression in an organism during an infection remains unclear. More studies are needed to better understand how AMPs act singly or in a mixture with other AMPs *in vivo*. For example, if the interactions between AMPs modify their spectrum of action. Alternatively, it could be proposed that the simultaneous expression of AMPs of the same family offers a certain degree of redundancy in the AMP cocktail. Such a redundancy could be adaptive if it could make the insect host resilient to the impairment of the activity of one or several AMPs during an interaction with a pathogen. Here we use gene knockdowns to study the *in vivo* effects of combinations of *T. molitor* Attacins in response to *P. entomophila* infection.

We use the mealworm beetle *T. molitor* as a model host to gain insight into the host-pathogen interaction upon bacterial infection and to uncover the importance combinations of Attacins. Previous studies have highlighted that antimicrobial activities of *T. molitor* AMPs *in vitro* do not reflect activities *in vivo*. There are several possible reasons for this such as synergisms among AMPs and local pH and salt concentration (3, 25). To date, there are five identified families of AMPs in *T. molitor* including the *Tenecin* (25, 26), *Attacin* (22), *Defensin* (27), *Coleoptericin* (28), *Cecropin* (29), and *Thaumatin-like protein* families (30). Some members of these families are named *Tenecins* for historical reasons.

*P. entomophila* is an entomopathogenic, Gram-negative bacterium that stimulates the induction of the immune deficiency (Imd) pathway in adult and larval *D. melanogaster* both systemically and locally in the gut following digestion. This results in the production of AMPs (31). *P. entomophila* has been shown to be highly pathogenic in a number of insect species, but is benign towards plants, suggesting that *P. entomophila* is a useful model to study host-pathogen interactions and a potential candidate for biocontrol agents (32). Previous studies of local and systemic innate immune responses in *D. melanogaster* and *Drosophila suzukii* after *P. entomophila* infection further support this conclusion (32–34):

We here knockdown members of one AMP family *in vivo* for the first time, investigating the effect of knocking down individual AMPs and their combinations on host response to infection with *P. entomophila*. We address whether depletion of several members of the Attacin gene family in *T. molitor* (*TmAttacins*) influences host survival and bacterial load, taking advantage of RNAi to provide single-, double-, and triple-knockdown of the Attacin family genes.

## 2 Materials and methods

### 2.1 Beetle rearing

Early instar mealworms were purchased from a commercial supplier (Reptile Food Handels-u. Zucht GmbH, Berlin, Germany). A group of 500 larvae were maintained in plastic containers (20 cm × 20 cm × 9 cm). They were fed wheat bran *ad libitum* and kept in the dark at 25 ± 3°C. Larvae were supplemented with a piece of fresh apple every two days as a source of moisture and nutrition. Every 48 h pupae were collected and sexed using a dissecting microscope (ZEISS stereo microscope STEMI 305). Previous studies have shown sexual dimorphism in immune function in *T. molitor*. Therefore, to reduce variation in survival and/or immune traits’ expression due to sex, only females were retained for experiments. These females were kept under the controlled conditions from eclosion onwards (35). All experimental individuals were 9-11 days-old and weighed 0.11-0.14 g.

### 2.2 Experimental design

To examine whether *P. entomophila* infection influences the expression levels of three *TmAttacin* genes, *T. molitor* females were exposed to either PBS or *P. entomophila* in PBS and expression changes in the Attacin genes confirmed *via* qRT-PCR. Following this confirmation, single- (*TmAtt1a*, *TmAtt1b*, and *TmAtt2*), double- (*TmAtt1a-Att2* and *TmAtt1b-Att2*), and triple-knockdowns (*TmAtt1a-Att1b-Att2*) were performed using RNAi. Double-stranded enhanced green fluorescent protein (ds*EGFP*) was injected as a negative control for the nonspecific effects of dsRNA. In each combination or single knockdown (KD) experiment, we needed to determine the time point at which the depletion in transcripts of our gene (s) of interest compared to ds*EGFP* was significant in *T. molitor* females. Therefore, efficiency of RNAi target gene suppression was evaluated in adult females collected from dsRNA- and ds*EGFP*-treated beetles. Based on these data, we selected the time points for *P. entomophila* infection. No infections were carried out before wounds from the knockdown injection had sufficiently healed. The immune tissues (haemolymph, fat body, gut, and Malpighian tubules) were dissected from infected beetles of all treatments for the first three days following infection and the survival of a second set of beetles was monitored daily for 35 days in parallel. A previous study on *S. aureus*-infected *T. molitor* females suggests that this time window captures most of the mortality caused by the infection without being confounded by senescence (3).

The beetles used in the survival assays were assigned to one of the following treatments: eitheruntreated control (designated as full control) or one of the three dsRNA-treated groups, ds*EGFP* (negative control) or gene (s) of interest-dsRNA (single-, double-, and triple-knockdown) groups. Each of these groups were divided into two subgroups, injected with PBS (control) or *P. entomophila* (**Figure S1**).

For bacterial load assays, colony forming units (CFU) were recovered from dsRNA-treated beetles only, namely ds*EGFP* and dsRNA (target gene (s) following *P. entomophila* infection (**Figure S1**).

### 2.3 Bacterial culture and injections

*P. entomophila* L48 (provided by Alexandro Rodríguez-Rojas) was cultured from 50% glycerol frozen stock at 28 ± 2°C in ten mL of Luria-Bertani (LB) broth overnight. 100 μL of overnight culture was harvested and reincubated in ten mL LB broth for a further 2 h under the same conditions. Cultures were interrupted when an optical density of 0.5 at 600 nm (OD_600_) was reached, measured by spectrophotometer (Ultrospec 10, Amersham Biosciences). The subcultured bacteria were concentrated by centrifugation and the cell pellet was washed three times with phosphate buffered saline (PBS) and then was resuspended. Based on the OD600, the culture was adjusted to 1 × 10^4^ cells/µL.

Infections were performed by injecting dsRNA-treated adults in the intersegmental membrane, between the fourth and fifth abdominal sternites, parallel to the anterior-posterior axis with a capillary needle filled with 5 μL of 1 × 10^4^ cells/µL of *P. entomophila*. Based on preliminary experiments, this infection concentration was likely to yield progressive mortality over time (**Figure S2**), allowing us to detect the effect of the various knockdowns of *TmAttacin* genes. After treatment, beetles were kept in cohorts of 15 individuals.

### 2.4 Gene expression analysis of *TmAttacin* genes upon *Pseudomonas entomophila* infection

In *T. molitor*, it was shown that last-instar larvae induce *TmAtt1a* and *-1b* in response to *E. coli*, suggesting the anti-Gram-negative activity of *TmAttacin* family (22). Thus, to elucidate whether this effect is also elicited by *P. entomophila*, we examined changes in the transcriptional abundance of *TmAttacin1a*, *TmAttacin1b*, and *TmAttacin2* following infection. To do so, we injected *P. entomophila* into female beetles (9-11 days-old) and measured changes in the expression patterns in our genes of interest using relative quantitative PCR (qRT-PCR). Four individual beetles were pooled together and homogenized with a pestle in liquid nitrogen before being transferred to 2 mL microcentrifuge tubes (Safe-Lock tubes, Eppendorf). Total mRNA was extracted using the Quick-RNA Tissue/Insect Microprep Kit (ZYMO Research Europe GmbH), following the manufacturer’s instructions. The resulting mRNA was stored at -80°C until being used for qRT-PCR. qRT-PCR was performed using Power SYBR™ Green RNA-to-CT™ 1-Step Kit (Applied Biosystems TM) with extracted mRNA and specific primers as listed in **Table S1**. The PCR amplification conditions were as follows: 95 °C for 5 min, followed by 40 cycles at 95 °C for 15 s and 60 °C for 30 s. Two technical replicates were carried out for each gene of interest along with the housekeeping gene *T. molitor 60S ribosomal protein L27a* (*TmL27a*) and averaged for each gene. The mean Ct of the AMP gene of interest was normalized using the mean Ct of *TmL27a* by calculating the mean Ct *TmL27a* - mean Ct of the gene of interest (delta Ct). Gene expression levels were expressed as 2^ (delta-delta Ct) (36).

### 2.5 cDNA synthesis and generation of double-stranded RNA

To silence target gene expression by RNAi, total RNA was extracted (Direct-zol RNA Miniprep Plus Kits, ZYMO Research) from the late instar larvae showing the highest enrichment of *TmAttacin1a* (GenBank accession No. MF754109), *TmAttacin1b* (MF754110), *TmAttacin2* (MF754108) (22) (**Figures S3–S5**), then cDNA fragments corresponding to each gene were produced using RevertAid™ Premium First Strand-cDNA-Synthese kit according to the manufacturer’s instructions. The cDNA was used as a template to amplify the fragments by PCR (KAPA2G Fast ReadyMix PCR Kit, KAPA Biosystems), following gene-specific primers tagged with a T7-promotor sequence in both 3’ and 5’ ends (**Table S1**). The PCR conditions were as follows: 95 °C for 2 min, followed by 30 cycles of denaturation at 95 °C for 20 s, annealing at 56 °C for 30 s, and extension at 72 °C for 5 min. A 508 bp PCR product of the Enhanced Green Fluorescent Protein (EGFP) gene derived from the plasmid (pGEM T-easy-GFP, Promega) was similarly amplified and used as a control for dsRNA (**Table S1**). After checking the length of the amplicons by running them on a 2% agarose gel, the PCR products were cleaned up using a kit (PCR/DNA Clean-Up DNA Kit, Roboklon). Using the resulting amplicons as template, *in vitro* transcription was carried out using a kit (HighYield T7 RNA Synthesis Kit, Jena Bioscience) as per the manufacturer’s recommendations. Next, the synthesized dsRNA was washed, and the RNA pellet was resuspended in nuclease-free water and kept at -20°C until further use.

### 2.6 Knockdown efficiency assessment and host survival assay

To address the individual and combined functional importance of *TmAttacin* family genes in *T. molitor* immunity *in vivo*, we selectively silenced one, two or three representative genes by RNAi. In the case of single-knockdowns (*TmAtt1a*, *TmAtt1b*, and *TmAtt2*), adult females were injected with 1000 ± 100 ng of dsRNA (500 μg/μL in 2 μL), for double-knockdowns 2000 ± 100 ng (1000 μg/μL in 2 μL) (*TmAtt1a-Att2* and *TmAtt1b-Att2*), and for triple-knockdown (*TmAtt1a-Att1b-Att2*) 3000 ± 100 ng of dsRNA in 6 µl total volume of nuclease-free water (i.e., circa 1000 ng for each representative genes). The same concentration of ds*EGFP* was injected for control insects, giving three control groups for single-dose, double-dose, and triple-dose controls, respectively.

To confirm RNAi efficiency of target genes, total RNA was extracted (n=4, pools of four adults per day) for single- (at first week and 14-day), double- (3-, 4- or 5-day), and triple-knockdown (4- and 5-day) at different time points post-exposure and then qRT-PCR carried out as described in section 2.4.

Next, to evaluate the viability of *T. molitor* females exposed to bacteria after silencing the target genes, there were six treatments in each set (single-, double-, and triple-knockdown) namely, full control - PBS, full control - P*. entomophila*, ds*EGFP* - PBS, ds*EGFP* - *P. entomophila*, dsRNA - PBS, dsRNA - P*. entomophila*. Survival assays were replicated twice in a separate set of beetles and bacterial cultures.

### 2.7 Cultivation and quantification of bacterial survival

To address whether *P. entomophila* successfully survive in the *T. molitor* tissues or are killed by the host immune system, the immune tissues of *TmAttacin* knockdown and ds*EGFP* beetles were dissected on days 1, 2, and 3 post-infections. Sampling was stopped at this time point due to a high number of dead beetles, which led to a reduced sample size. We collected haemolymph, fat body, gut, and Malpighian tubules of each *T. molitor* female before pooling and homogenizing them in a 2 mL microcentrifuge tube (Safe-Lock tubes, Eppendorf) containing 250 μL of LB broth and two stainless steel beads (Ø 3 mm, Retsch) on ice. Collected tissues were homogenized at a frequency of 30 Hz for 20 seconds using a tissue homogenizer (Mill MM400, Retsch) before being centrifuged at 420 × g for 1 minute at 4 °C. Homogenates from each beetle were added into 180 μL of PBS and then serially diluted 1:10 to 1:10^5^ (**Table S2**). Isolated *P. entomophila* were cultured on LB medium containing 1000 μg/mL of ampicillin for 24 h at 28°C (37). Four drops (5 μL) per insect were counted as replicates and averaged. We injected a subset of KD and control beetles with PBS to check whether we could retrieve colonies from their bodies: no colonies were recovered from uninfected dsRNA-treated females (n =15 beetles each per treatment).

### 2.8 Statistics

All data were analyzed using the R software version 4.1.2 (38).

The survival of *T. molitor* females was analyzed with a Cox model for proportional hazards (checked with ‘coxzph’ function), with host survival as a response variable, and with knockdown treatments and infection treatments as explanatory variables along with experimental replicate as a random factor (package ‘coxme’) (39). When relevant, post-hoc comparisons were performed by looking at contrasts between treatment levels in the summary of the optimal model.

We analyzed the *P. entomophila* bacterial load retrieved from the beetles, measured as the number of CFU obtained on agar plates, with a generalized linear model fitted for a negative binomial distribution (‘glm.nb’ function of the ‘MASS’ package (40). CFU counts were the response variable, whereas time (days 1, 2 or 3) and knockdown treatments were the explanatory variables. *Post hoc* comparisons are performed by comparing the overlap of 95% confidence intervals (95%CI) around the estimates of the optimal model. The differences were considered significant if two 95%CI error bars overlap by no more than about half of their length (41). The effect plots for *post hoc* comparisons are presented in **Figure S17**.

In all analyses, we built the most complex model including all explanatory variables and interactions between them, as well as all the possible nested models and the null model. We then selected the optimal model based on the comparison of Akaike’s Information Criterion (AIC). Models with the lowest AIC - or lowest degree of complexity for an equivalent AIC - were retained as the optimal model. Models comprised within a delta AIC of two were considered equivalent (42).

Since different experiments on single-, double-, or triple-knockdowns had a different amount of ds*EGFP* as a negative control, the results yielded by different knockdown treatments were not directly comparable. However, we are able to circumvent this experimental feature by comparing the effect sizes of the knockdowns relative to their respective control treatments, both for bacterial load and host survival. This approach allows for a robust comparison of the effects of single, double, and triple knockdowns of the *TmAttacin* genes expression between experiments, as shown in Zanchi et al. (2017) (3). Therefore, to compare the effects of various knockdown treatments, we analyzed the effect size differences between each knockdown treatment and its respective control (ds*EGFP*) which is shown in the last section of the results.

In the case of the survival analysis, we used the Hazard Ratio (HR), obtained from the summary of the optimal model of each experiment, as a measure of effect size. The HR consists of the ratio of the hazard rates of the knockdown treatment to the respective ds*EGFP* control within each experiment, and represents the relative risk of dying of the knockdown versus ds*EGFP* (43).

In the case of the CFU counts, we chose Hedge’s g as an effect size, which like Cohen’s d describes the standardized mean difference of an effect but is further corrected for small sample sizes. It is calculated as the difference between the mean CFU counts of the knockdown treatment and the respective ds*EGFP* control, divided by the weighted pooled standard deviation for the two treatments (44).

We then plotted these parameters and the 95% confidence intervals around the effect for each knockdown experiment. An overlap of the confidence intervals around the HR with 1 indicates no significant difference between knockdown treatment and control in the risk of dying. A HR greater than 1 indicates a higher risk of dying, whereas a HR lower than 1 indicates a lower risk of dying in the knockdown treatment compared to control. Similarly, an overlap of the confidence intervals around Hedge’s g with 0 indicates no effect of the knockdown treatment compared to its respective ds*EGFP* control, a value greater than 0 indicates a positive effect, whereas a value smaller than 0 indicates a negative effect of the knockdown on CFU count compared to control. Both the HR and Hedge’s g were calculated using the package “effectsize” (45).

## 3 Results

### 3.1 *Tenebrio molitor Attacin* genes induction and RNAi-mediated gene knockdown

The result of the induction experiment was consistent with previous study that shows that bacterial challenge led to a significant increase in transcription of *TmAttacin1a*, *-1b*, and *-2* compared to PBS-injected controls (**Figure S6**) (22). Based on this we carried out three sets of experiments with *P. entomophila* infection, in which single (*TmAtt1a*, *TmAtt1b*, and *TmAtt2*), pairs of (*TmAtt1a-Att2* and *TmAtt1b-Att2*), and all three (*TmAtt1a-Att1b-Att2*) AMP genes were silenced.

In ds*TmAtt1a*-treated females, we observed a consistent decrease of *TmAtt1a* mRNA transcript, ranging from 4-day to 14-day post-exposure. Accordingly, the 4-day RNAi treatment was selected as a time point for bacterial challenge (**Figure S7**). For the series of experiments on *TmAtt1b-*silenced females were challenged 3-days post-RNAi treatment, at which point relative expression of *TmAtt1b* transcript was successfully suppressed (**Figure S8**). Similarly, we challenged *TmAtt2*-silenced females at 3-days post knockdown treatment (**Figure S9**). In ds*TmAtt1a-Att2*-treated beetles, females were infected at 4-days post dsRNA injection (**Figure S10**), while they were challenged at 3-day in ds*TmAtt1b-Att2*-treated beetles (**Figure S11**). Downregulation of the relative expression of *TmAtt1a* in ds*TmAtt1a-Att1b*-treated beetles was not successful (**Figure S12**).

Next, to tackle the effect of representative genes on each other, the transcription levels of all *TmAttacin* in ds*TmAtt1a*- and ds*TmAtt1b-*treated beetles were separately assessed. As can be seen in **Figures S13, 14**, silencing *TmAtt1a* (4-day) had no effect on expression level of either *TmAtt1b* or *TmAtt2*, similarly, *TmAtt1b* (3-day) knockdown did not influence transcript levels of *TmAtt1a* or *TmAtt2*. Based on the double-knockdown results, we altered the volume of representative genes to successfully silence all three genes. In triple-knockdown beetles we accounted for the reduced expression of *TmAtt1a*, *TmAtt1b*, and *TmAtt2* after RNAi treatment of each gene separately. Therefore, we expected that 4-day post-exposure females would be optimal for challenging, which was confirmed *via* qRT-PCR (**Figures S15, S16**).

### 3.2 Single knockdown of members of the *TmAttacin* gene family

#### 3.2.1 Knockdown of *TmAttacin1a* does not influence host survival and bacterial load

**Figure 1:**
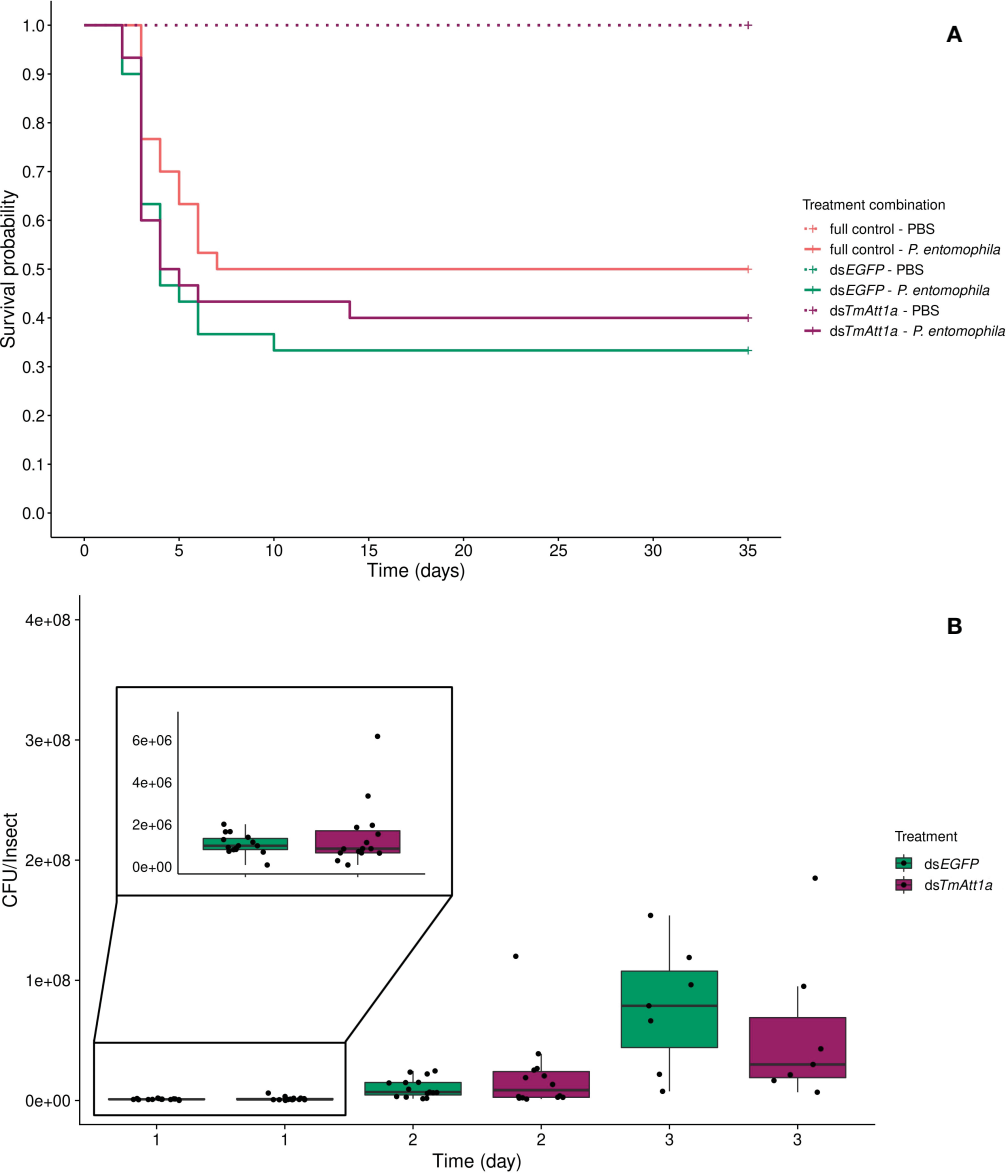
**(A)** Effect of *TmAttacin1a* (*TmAtt1a*) knockdown on survival of *Pseudomonas entomophila* infected *Tenebrio molitor* females over 35 days. Two independent experiments each performed on 15 females per treatment group. Each dsRNA-treated or dsRNA-untreated group was infected with *P. entomophila* on day 4 after dsRNA treatment based on the data on knockdown efficiency (**Figure S7**). Pink dashed line: Full control-PBS; Turquoise dashed line: ds*EGFP* - PBS; Purple dashed line: ds*TmAtt1a* - PBS; Pink line: Full control - P*. entomophila*; Turquoise line: ds*EGFP* - P*. entomophila*; Purple line: ds*TmAtt1a* - P*. entomophila*. **(B)** Bacterial load in ds*TmAtt1a*-silenced *T. molitor* challenged with *P. entomophila* at 1-, 2-, and 3-day post-infections. The colony-forming units (CFU) recovered from tissues of *T. molitor* females from ds*TmAtt1a* (purple) and ds*EGFP* (control, turquoise). In the box plots, the lower (first) quartile is the closest boundary to zero, the line within the box marks the median (second quartile), and the upper (third) quartile. Each dot represents the CFU count in an individual beetle. The bars represent the 1.5 interquartile. Sample sizes are given in **Table S2**.

We tested whether depleting *TmAtt1a* transcripts could increase mortality of *T. molitor* females following challenge with *P. entomophila*. To do so, we infected beetles at 4-days post-RNAi treatment and monitored survival daily for 35 days. We observed that PBS-injected insects (ds*TmAtt1a*-PBS, ds*EGFP*-PBS, and full control-PBS) showed no death over 35 days, therefore we did not include them in the analysis, but presented them in the figures. We did not find an effect of knockdown treatment in *P. entomophila* injected beetles, since the survival of infected-ds*TmAtt1a* (ds*TmAtt1a-P. entomophila*, ds*EGFP-P. entomophila*, and full control-*P. entomophila*) was not significantly different from the control treatments (X²_3.177_ = 1.2, *p* = 0.55) (**Figure 1A**).

Consistent with this observation, the single knockdown of *TmAtt1a* did not influence *P. entomophila* load in the bodies of the beetles, neither in interaction with time (time × KD treatment: X²_2.72 _= 2.667, *p* = 0.26) nor as a simple effect (X²_1.72 _= 1.17e^-05^, *p* = 1). CFU counts increased over 3 days (time: X²_2.72 _= 173.38, *p* < 0.0001) (**Figure 1B**, **S17A**).

#### 3.2.2 Knockdown of *TmAttacin1b* has an effect on survival and bacterial load

**Figure 2:**
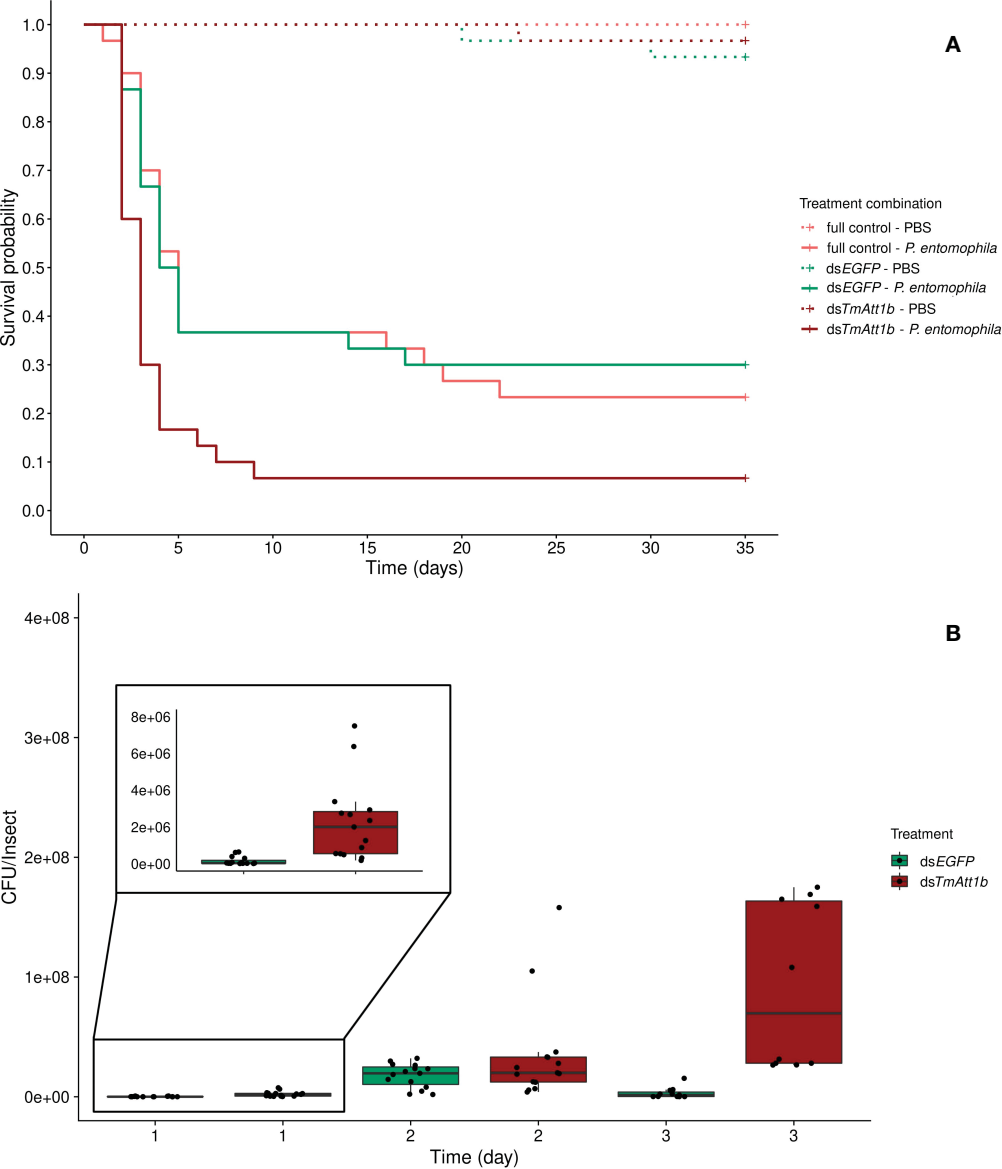
**(A)** Effect of *TmAttacin1b* (*TmAtt1b*) knockdown on survival of *Pseudomonas entomophila* infected *Tenebrio molitor* females over 35 days. ds*EGFP* was injected as a negative control for the nonspecific effects of dsRNA and the third group includes dsRNA untreated control. Two independent experiments each performed on 15 females per treatment group. Each dsRNA-treated or dsRNA-untreated groups were infected with *P. entomophila* on day 3 after dsRNA treatment based on the data on knockdown efficiency (**Figure S8**). Pink dashed line: Full control-PBS; Turquoise dashed line: ds*EGFP* - PBS; Burgundy dashed line: ds*TmAtt1b* - PBS; Pink line: Full control - P*. entomophila*; Turquoise line: ds*EGFP* - P*. entomophila*; Burgundy line: ds*TmAtt1b* - P*. entomophila*. **(B)** Bacterial load in ds*TmAtt1b*-silenced *T. molitor* challenged with *P. entomophila* at 1-, 2-, and 3-day post-infections. The colony-forming units (CFU) recovered from tissues of *T. molitor* females from ds*TmAtt1b* (burgundy) and ds*EGFP* (control, turquoise). In the box plots, the lower (first) quartile is the closest boundary to zero, the line within the box marks the median (second quartile), and the upper (third) quartile. Each dot represents the CFU count in an individual beetle. The bars represent the 1.5 interquartile. Sample sizes are given in **Table S2**.

The optimal model did not include an effect of the interaction between knockdown (KD) and infection treatments on beetle survival (KD. treatment × inf. treatment: X²_5.175 _= 3.41, *p* = 0.18). However, it is important to note that the mortality in the PBS injected beetles is negligible across all knockdown treatments (only 3 dead beetles) (**Figure 2A**).

Both knockdown and infection treatment influenced beetle survival as simple effects (KD. treatment: X²_2.178_ = 13.22, *p* = 0.0013; infection treatment: X²_2.178_ = 46.85, *p* < 0.0001). We observed a strong decrease in survival of beetles after *P. entomophila* challenge (**Figure 2A**). There was a decrease in survival when beetles that were treated with ds*TmAtt1b* and subsequently infected were compared to those treated with ds*EGFP* and infected (ds*TmAtt1b*/ds*EGFP*: z = 3.07, *p* = 0.0021) while no difference in survival of infected full control and infected ds*EGFP* was found (Full control/ds*EGFP*: z = -0.07, *p* = 0.95).

The dynamics of the bacterial load were significantly different in ds*TmAtt1b*-treated beetles compared to ds*EGFP* beetles over time (time × KD treatment: X²_2.80 _= 23.35, *p* = 8.512e^-06^). While the density of *P. entomophila* in ds*EGFP*-treated females increased between 1-day and 2-day post- infection, bacterial load of ds*TmAtt1b*-treated beetles showed higher densities starting from 1-day post infection. CFU counts of *P. entomophila* were similar between *TmAtt1b* knockdown treatment and control at 2-days post-infection. Interestingly, bacterial load of ds*EGFP*-treated females significantly decreased between 2-days and 3-days post-infection, whereas it remained similarly high between 2- and 3-days in ds*TmAtt1b*-treated beetles (**Figures 2B**, **S17B**).

#### 3.2.3 Knockdown of *TmAttacin2* influences survival and bacterial load

**Figure 3:**
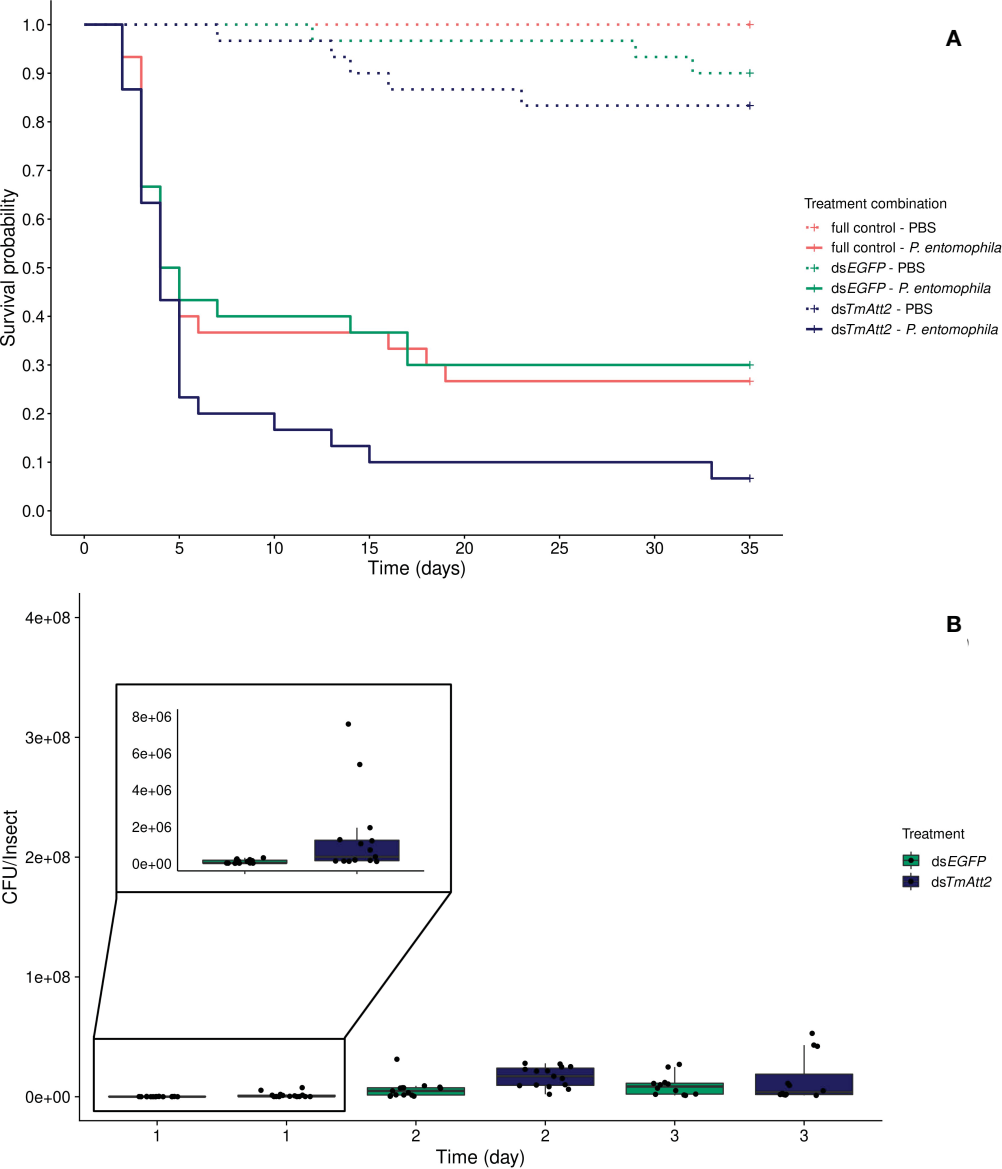
**(A)** Effect of *TmAttacin2* (*TmAtt2*) knockdown on survival of *Pseudomonas entomophila* infected *Tenebrio molitor* females over 35 days. ds*EGFP* was injected as a negative control for the nonspecific effects of dsRNA and the third group includes dsRNA untreated control. Two independent experiments each performed on 15 females per treatment group. Each dsRNA-treated or dsRNA-untreated groups were infected with *P. entomophila* on day 3 after dsRNA treatment based on the data on knockdown efficiency (**Figure S9**). Pink dashed line: Full control-PBS; Turquoise dashed line: ds*EGFP* - PBS; Navy blue dashed line: ds*TmAtt2* - PBS; Pink line: Full control - P*. entomophila*; Turquoise line: ds*EGFP* - P*. entomophila*; Navy blue line: ds*TmAtt2* - P*. entomophila*. **(B)** Bacterial load in ds*TmAtt2*-silenced *T. molitor* challenged with *P. entomophila* at 1-, 2-, and 3-day post-infections. The colony-forming units (CFU) recovered from tissues of *T. molitor* females from ds*TmAtt2* (navy blue) and ds*EGFP* (control, turquoise). In the box plots, the lower (first) quartile is the closest boundary to zero, the line within the box marks the median (second quartile), and the upper (third) quartile. Each dot represents the CFU count in an individual beetle. The bars represent the 1.5 interquartile. Sample sizes are given in **Table S2**.

Similarly, to the KD of *TmAtt1b*, we found no interaction between knockdown and infection treatment on beetle survival (KD. treatment × infection treatment: X²_5.120 _= 4.98, *p* = 0.083). Instead, both the knockdown and the infection treatments affect survival of beetles as simple effects. Again, *TmAtt2*-silenced treatments showed a decreased survival compared to the ds*EGFP* control (KD. treatment: X²_3.115 _= 7.11, *p* = 0.029; ds*TmAtt2*/ds*EGFP*: z = 2.17; *p* = 0.03), whereas the survival of ds*EGFP* beetles does not differ compared to full control (Full control *vs*. ds*EGFP*: z = -0.07, *p* = 0.95) (**Figure 3A**). Moreover, as expected, infection by *P. entomophila* led to high mortality in beetles (infection treatment: X²_3.115 _= 111.02, *p* < 0.0001).

The development of the bacterial load over time is different in *TmAtt2*-silenced beetles compared to the control (time × KD treatment: X²_5.83 _= 15.47, *p* = 0.00042). While CFU counts increased over 3 days in the control, as seen in the previous knockdowns, they already reached higher counts 1-day post-injection in ds*TmAtt2*-treated group compared to ds*EGFP* control. *TmAtt2*-silenced beetles increased and remained stable over the first two days post infection, whereas the control reached similar CFU counts at 3-days post challenge (**Figures 3B**, **S17C**).

### 3.3 Double Knockdown of *TmAttacin* family

#### 3.3.1 Knockdown of *TmAttacin1a* and *TmAttacin2* does not influence host survival but influences bacterial load

**Figure 4:**
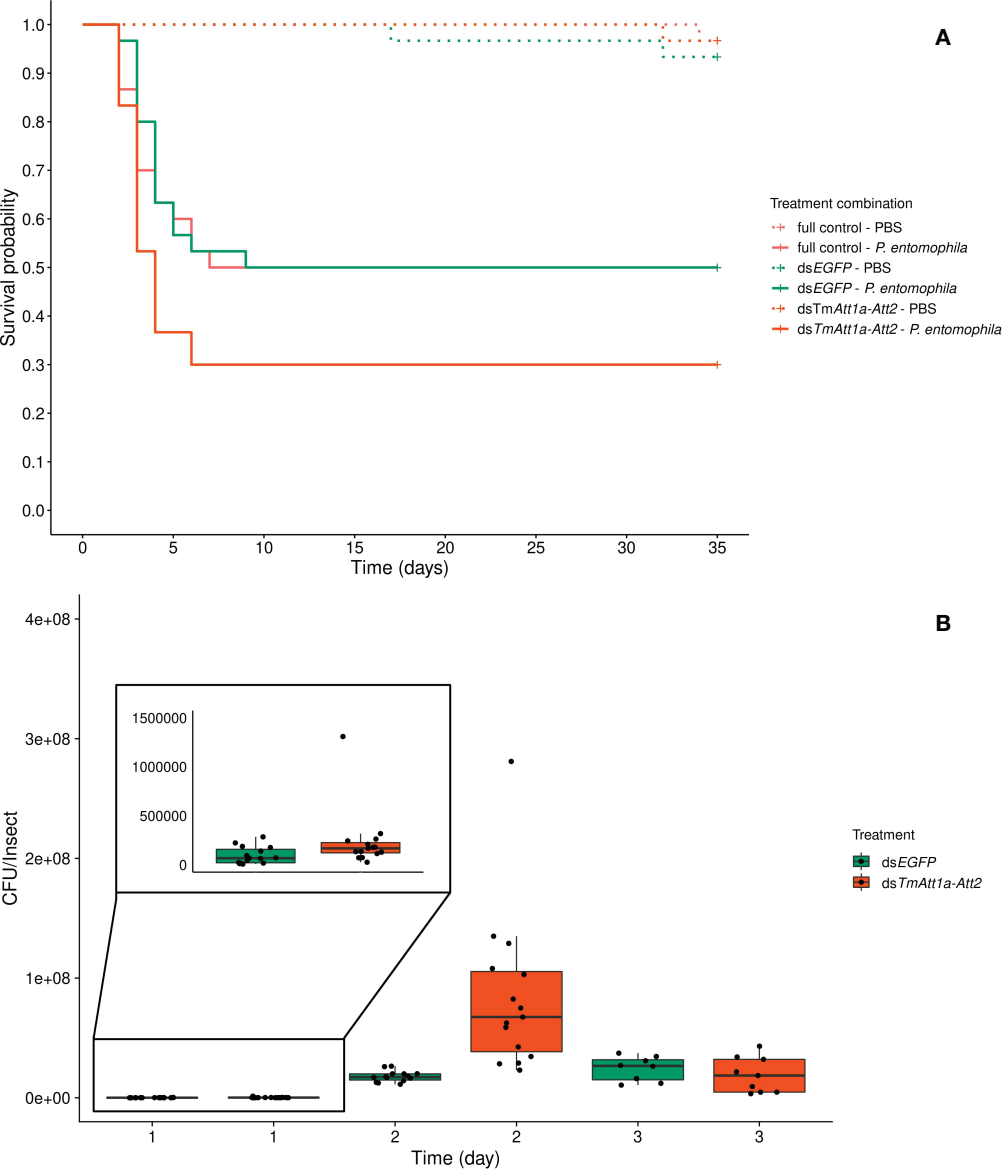
**(A)** Effect of *TmAttacin1a-2* (*TmAtt1a-Att2*) double-knockdown on survival of *Pseudomonas entomophila* infected *Tenebrio molitor* females over 35 days. ds*EGFP* was injected as a negative control for the nonspecific effects of dsRNA and the third group includes dsRNA untreated control. Two independent experiments each performed on 15 females per treatment group. Each dsRNA-treated or dsRNA-untreated groups were infected with *P. entomophila* on day 4 after dsRNA treatment based on the data on knockdown efficiency (**Figure S10**). Pink dashed line: Full control-PBS; Turquoise dashed line: ds*EGFP* - PBS; Orange dashed line: ds*TmAtt1a-Att2* - PBS; Pink line: Full control - P*. entomophila*; Turquoise line: ds*EGFP* - P*. entomophila*; Orange line: ds*TmAtt1a-Att2* - P*. entomophila*. **(B)** Bacterial load in ds*TmAtt1a-Att2*-silenced *T. molitor* challenged with *P. entomophila* at 1-, 2-, and 3-day post-infection. The colony-forming units (CFU) recovered from tissues of *T. molitor* females from ds*TmAtt1a-Att2* (orange) and ds*EGFP* (control, turquoise). In the box plots, the lower (first) quartile is the closest boundary to zero, the line within the box marks the median (second quartile), and the upper (third) quartile. Each dot represents the CFU count in an individual beetle. The bars represent the 1.5 interquartile. Sample sizes are given in **Table S2**.

The double KD of *TmAtt1a* and *TmAtt2* treatment had no effect on survival, neither in interaction with the infection treatment (KD. treatment × infection treatment: X²_5.175_ = 1.23; *p* = 0.54) nor as a simple effect (KD. treatment: X²_2.173_ = 2.04, *p* = 0.36). Only the infection treatment was retained in the best model, where the survival of adult females infected with *P. entomophila* was significantly lower (X²_1.174_ = 32.35, *p* < 0.0001) (**Figure 4A**).

For *P. entomophila* density inside the beetles, the best model retained an interaction between treatment and time (time × KD treatment: X²_5.75 _= 16.05, *p* = 0.00033). ds*EGFP*-treated beetles showed the same dynamics as described above, the CFU count increased between 1-day and 2-days post-infection and remained stable between 2- and 3-days post-infection. Double-knockdown of *TmAtt1a* and *TmAtt2* resulted in a higher number of CFU recovered from beetles starting from 1-day after challenge. Moreover, it reached a higher CFU count than the control at 2-days post infection and at 3-days decreased to a level similar to controls (**Figure 4B** and **S17D**).

#### 3.3.2 Double knockdown of *TmAttacin1b* and *TmAttacin2* does not influence host survival but influences bacterial load

**Figure 5:**
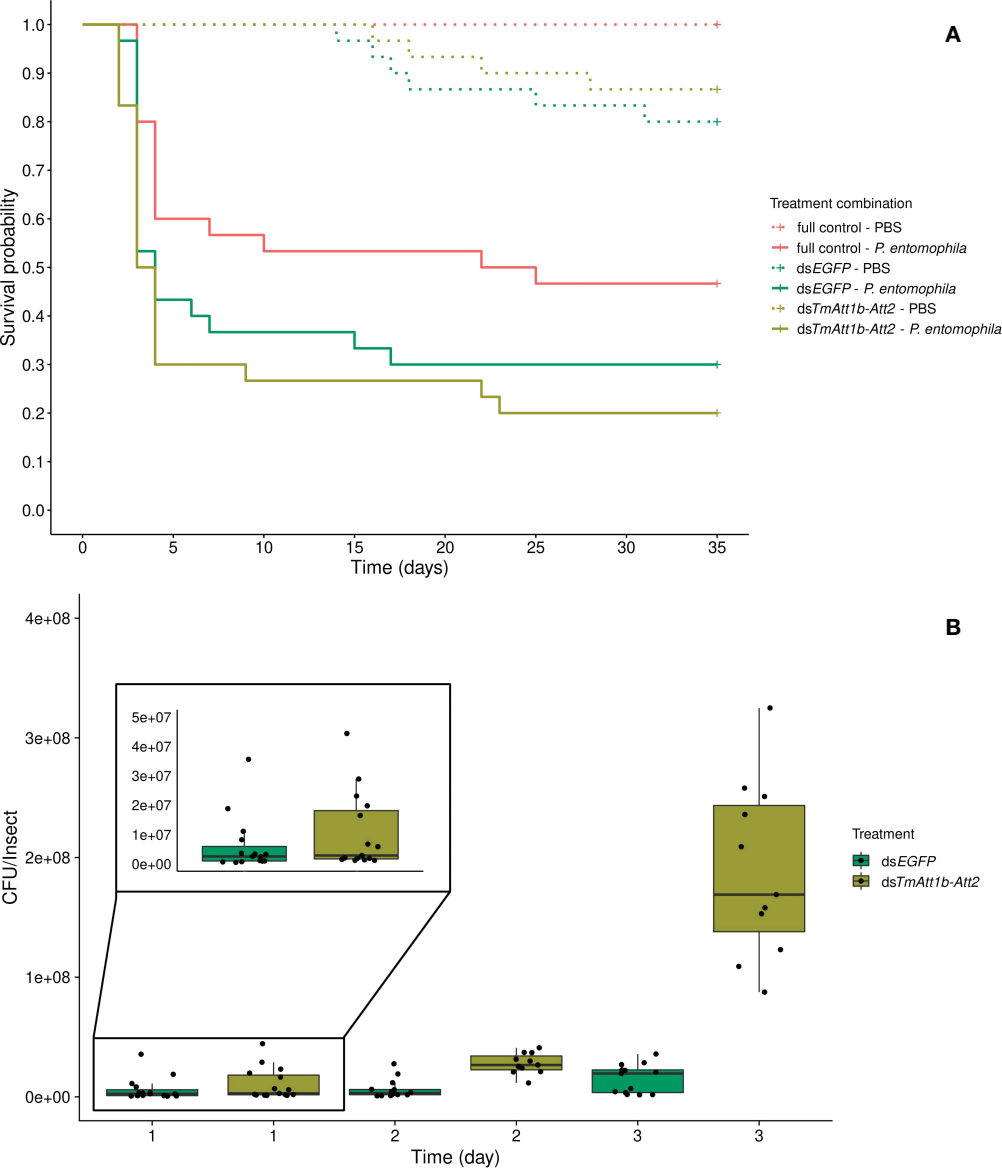
**(A)** Effect of *TmAttacin1b-2* (*TmAtt1b-Att2*) double-knockdown on survival of *Pseudomonas entomophila* infected *Tenebrio molitor* females over 35 days. ds*EGFP* was injected as a negative control for the nonspecific effects of dsRNA and the third group includes dsRNA untreated control. Two independent experiments each performed on 15 females per treatment group. Each dsRNA-treated or dsRNA-untreated groups were infected with *P. entomophila* on day 3 after dsRNA treatment based on the data on knockdown efficiency (**Figure S11**). Pink dashed line: Full control-PBS; Turquoise dashed line: ds*EGFP* - PBS; Olive green dashed line: ds*TmAtt1b-Att2* - PBS; Pink line: Full control - P*. entomophila*; Turquoise line: ds*EGFP* - P*. entomophila*; Olive green line: ds*TmAtt1b-Att2* - P*. entomophila*. **(B)** Bacterial load in ds*TmAtt1b-Att2*-silenced *T. molitor* challenged with *P. entomophila* at 1-, 2-, and 3-day post-infection. The colony-forming units (CFU) recovered from tissues of *T. molitor* females from ds*TmAtt1b-Att2* (olive green) and ds*EGFP* (control, turquoise). In the box plots, the lower (first) quartile is the closest boundary to zero, the line within the box marks the median (second quartile), and the upper (third) quartile. Each dot represents the CFU count in an individual beetle. The bars represent the 1.5 interquartile. Sample sizes are given in **Table S2**.

Despite a trend towards an interaction between KD and infection treatment (KD. treatment × infection treatment: X²_3.177_ = 5.185, *p* = 0.075), the best model is one in which beetle survival is explained by both simple effects of the infection (inf. treatment: X²_2.179 _= 50.46, *p* < 0.0001) and knockdown treatments (KD. treatment: X²_2.179_ = 9.859, *p* = 0.0072). As expected, *P. entomophila* infection decreased beetle survival. However, the effect of the KD treatment does not come from a lower survival of *TmAtt1b-Att2* compared to ds*EGFP* (ds*TmAtt1b-Att2*/ds*EGFP*: z = 0.64, *p* = 0.52). It is caused by the fact that beetles with no injection of dsRNA outlived both ds*EGFP* (full control/ds*EGFP*: z = -2.50, *p* = 0.013) and ds*TmAtt1b-Att2* (full control/ds*TmAtt1b-Att2*: z = -3.06, *p* = 0.002) (**Figure 5A**).

There was an interaction between KD treatment and time on the bacterial load recovered from the beetles (time × KD treatment: X²_5.79 _= 14.75, *p* = 0.00063). The CFU counts are similar at 1-day post-infection in both treatments but diverge at 2-days, where they reach higher concentrations in the KD. This effect persists at 3-days post-infection (**Figures 5B**, **S17E**). Therefore, the CFU counts observed higher in the double-knockdown treatment than in control at both 2-days and 3-days post injection.

### 3.4 Triple knockdown of *TmAttacin* family

The next question was how triple knockdown could influence survival of females upon *P. entomophila* infection, given that double knockdown did not reveal any significant difference in beetle survival (**Figures 4**, **5**).

#### 3.4.1 Effect of triple knockdown of *TmAttacin1a*, *TmAttacin1b*, and *TmAttacin2* on survival following infection

**Figure 6:**
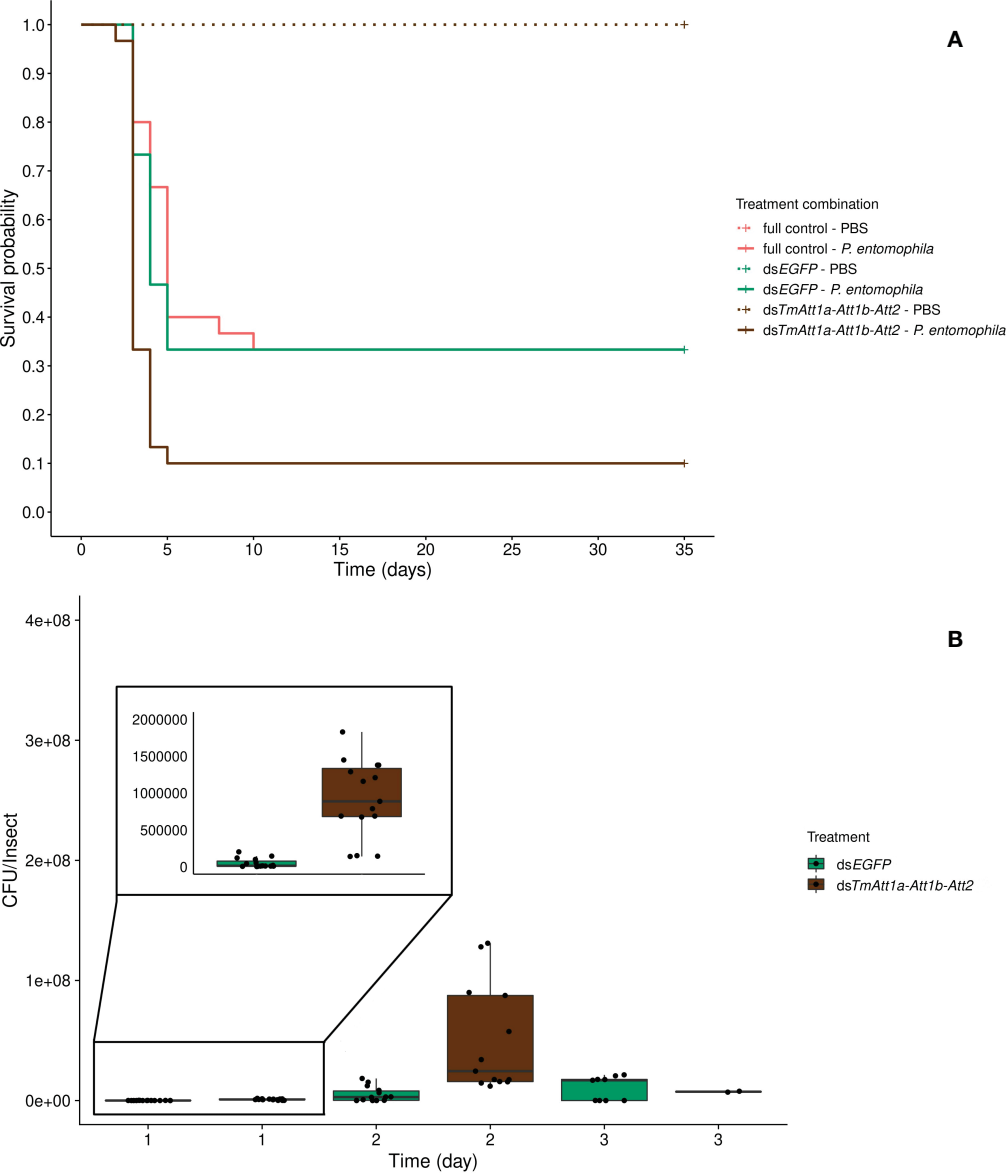
**(A)** Effect of *TmAttacin1a-1b-2* (*TmAtt1a-Att1b-Att2*) triple-knockdown on survival of *Pseudomonas entomophila* infected *Tenebrio molitor* females over 35 days. ds*EGFP* was injected as a negative control for the nonspecific effects of dsRNA and the third group includes dsRNA untreated control. Two independent experiments each performed on 15 females per treatment group. Each dsRNA-treated or dsRNA-untreated groups were infected with *P. entomophila* on day 4 after dsRNA treatment based on the data on knockdown efficiency (**Figure S15**). Pink dashed line: Full control-PBS; Turquoise dashed line: ds*EGFP* - PBS; Brown dashed line: ds*TmAtt1a-Att1b-Att2* - PBS; Pink line: Full control - P*. entomophila*; Turquoise line: ds*EGFP* - P*. entomophila*; Brown line: ds*TmAtt1a-Att1b-Att2* - P*. entomophila*. **(B)** Bacterial load in *dsTmAtt1a-Att1b-Att2*-silenced *T. molitor* challenged with *P. entomophila* at 1-, 2-, and 3-day post-infection. The colony-forming units (CFU) recovered from tissues of *T. molitor* females from *dsTmAtt1a-Att1b-Att2* (brown) and ds*EGFP* (control, turquoise). In the box plots, the lower (first) quartile is the closest boundary to zero, the line within the box marks the median (second quartile), and the upper (third) quartile. Each dot represents the CFU count in an individual beetle. The bars represent the 1.5 interquartile. Sample sizes are given in **Table S2**.

Beetles which were injected with PBS only (ds*TmAtt1a-Att1b-Att2*-PBS, ds*EGFP*-PBS, and full control-PBS) display no mortality over the time course of this experiment. We further carried out the survival analysis on *P. entomophila* injected beetles only, however PBS-injected treatments are displayed in the figures. We detected that knockdown treatment significantly affects the survival of *P. entomophila* injected beetles (KD. treatment:X²_2.87_ = 16.349, *p* = 0.00028). The triple knockdown of *TmAtt1a*, *TmAtt1b*, and *TmAtt2* by RNAi reduced the survival of females following *P. entomophila* infection (ds*TmAtt1a-Att1b-Att2*/ds*EGFP*: z = 3.22, *p* = 0.0013) while survival of ds*EGFP* beetles does not differ compared to full control beetles (full control *vs*. ds*EGFP*: z = -0.42, *p* = 0.68) (**Figure 6A**). Since no effect of the KD is detectable among PBS-injected beetles, but a strong effect exists in *P. entomophila*-injected beetles, there is an interaction between KD and infection treatments on beetle survival.

The optimal model does not include an interaction between time and knockdown treatment for bacterial loads (time × KD treatment: X²_3.67 _= 5.22, *p* = 0.074), however, the CFU counts are explained by both time (time: X²_2.67 _= 103.35, *p <*0.0001) and knockdown treatment (KD treatment: X²_1.67 _= 39.39, *p <*0.0001) as simple effects. *P. entomophila* load increased initially (1-day and 2-days post-infection) and remained stable at the last day in both treatments. CFU counts were overall higher in *TmAtt1a-Att1b-Att2*-silenced beetles than in the control (**Figures 6B**, **S17F**).

### 3.5 Relative effects of single, double, and triple knockdowns on survival and bacterial load

Since the different knockdown experiments have different controls, due to the different concentration of ds*EGFP*-injected, we normalized survival and pathogenic load of the KD beetles relative to their respective control, using effect size measures This approach can highlight potential interactions between AMPs, by showing that a multiple knockdown yields a higher effect than the sum of its single knockdowns, or on the contrary, by highlighting a lower effect of a multiple knockdown compared to single knockdowns. The following section summarizes the results of our experiments by calculating and presenting the effects of different knockdowns compared to their respective ds*EGFP* controls on the survival and the bacterial load of *T. molitor* females infected with *P. entomophila*.

**Figure 7:**
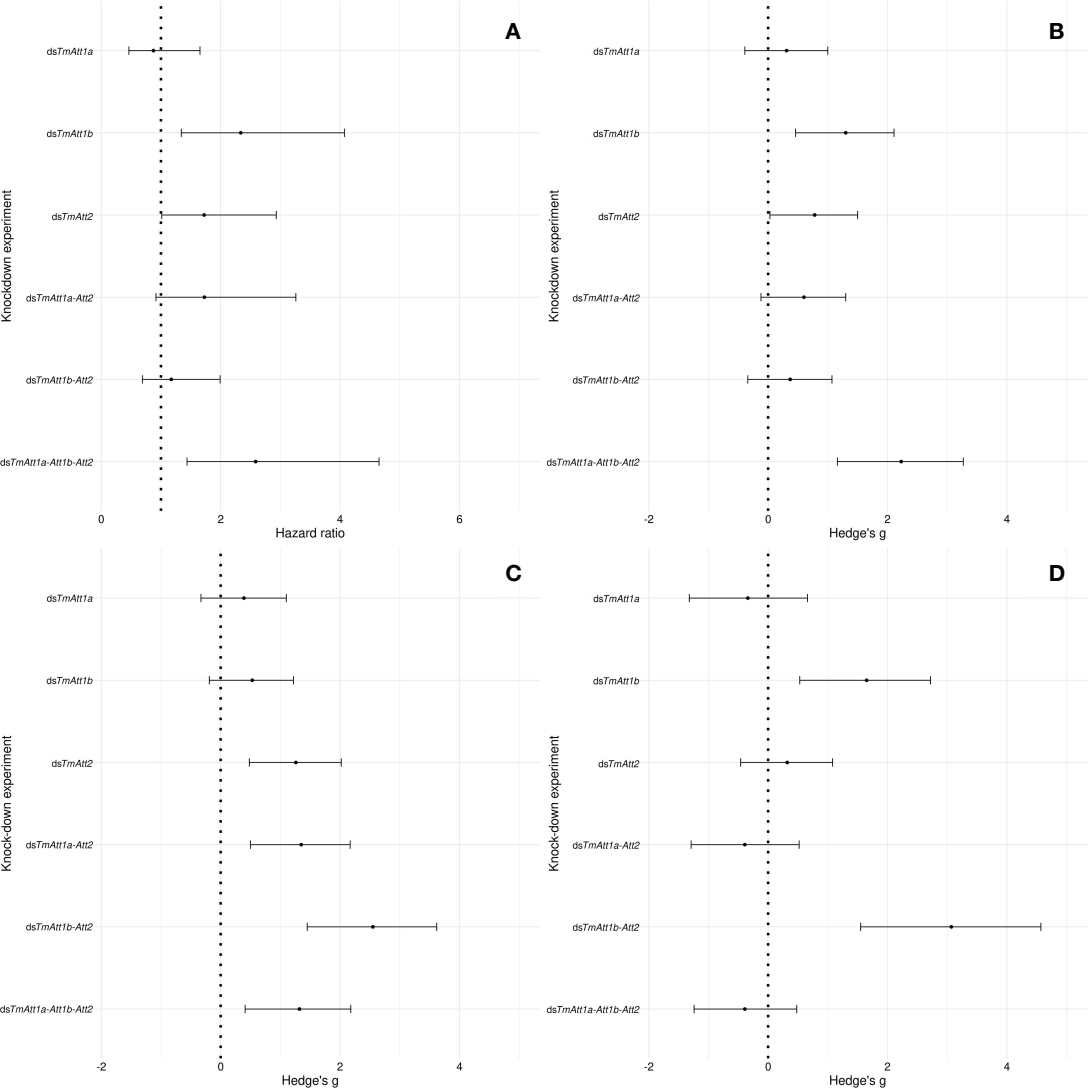
Forest plot representing the effects of the single, double, or triple knockdown of the AMP genes of interest on **(A)** survival of *Tenebrio molitor* females infected by *Pseudomonas entomophila* and **(B)** the number of *P: entomophila* CFU retrieved from females at 1-day, **(C)** 2-days, and **(D)** 3-days post-infection. In case of the survival, the effect size is calculated as the hazard ratio (HR) between knockdown and ds*EGFP* control groups **(A)**. In case of CFU counts **(B–D)**, it is calculated as the Hedge’s g between knockdown and ds*EGFP* control groups. Dots represent the HR and Hedge’s g, and the horizontal bars extend from the lower limit to the upper limit of the 95% confidence interval (95CI). An overlap of the 95CI with 1 (vertical line) means the knockdown has no effect on survival compared to control. A HR < 1 indicates a reduction in the hazard, whereas a HR > 1 indicates an increase in hazard In the knockdown compared to control, an overlap of the 95CI with 0 (vertical dotted line) indicates no significant effect of the knockdown treatments compared to control on CFU count, a positive value indicates a positive effect of the knockdown on CFU count, whereas a negative value indicates a negative effect of the knockdown on CFU count compared to control.

The differential dynamics of the effects of KD on bacterial load was not reflected in the survival curves, as shown by the hazard ratios (HR) (**Figure 7A**). Instead, there is a remarkable correlation between the plot of the HR of the survival analyses and the plot of the Hedge’s g of the various treatments 1-day post injection (**Figures 7A, B**). Indeed, hazard ratios of the various treatments are explained by Hedge’s g on CFU count of the same treatments at 1-day post-infection (linear model: Hedge’s g: F_1.4_ = 20.41, *p* = 0.01). This is not the case at 2- (F_1.4 _= 0.073, *p* = 0.8) and 3-days (F_1.4 _= 0.1, *p* = 0.77) post-infection.

Looking more closely at the effect sizes of the various knockdown treatments on CFU counts over time, we notice that the effect of single knockdowns is relatively constant over time, i.e., the 95CI around the models’ estimates overlap within each single-knockdown treatment between time points.

Knockdown of *TmAtt1a* did not have an effect on the CFU count of *P. entomophila* over 3 days (95CI overlaps zero at each time points, **Figures 7B–D**). Knockdown of *TmAtt2* had a larger effect at days 1 and 2 compared to the other single knockdowns, however, the effect disappeared at 3-days post-infection. Notably, a larger effect was observed in ds*TmAtt1b-* than in ds*TmAtt2-*treated females on day three. However, double-knockdown of *TmAtt1b-Att2* yielded a slightly lower effect than both their single-knockdowns on day one. The effects of *TmAtt2* and *TmAtt1b* knockdowns on CFU counts were dynamic over time.

The effect of double-knockdown of *TmAtt1b-Att2* gradually increased over 3 days, from showing no effects on day one to reaching the highest effect than any other single and double knockdown at 3-days post-infection. Interestingly, this observation does not follow the dynamics of the effects of these genes when they were separately knocked down, since the single-knockdown of both genes had an effect on day one.

Finally, the effect of the triple-knockdown on *P. entomophila* load was higher at 1-day post infection compared to other knockdown experiments but decreased on day two. Of note, the sample size dropped at 3-day post-infection because all the highly infected individuals had died (**Figures 7B–D**).

## 4 Discussion

AMPs as conserved molecules frequently combat pathogenic infections both synergistically and individually (12). Results of our study show that the knockdown of AMP genes in *T. molitor* either individually or in combinations of three AMP genes of the same family influences host survival following *P. entomophila* infection. *T. molitor* mortality is associated with bacterial load in host tissues, consistent with *Drosophila* studies (46, 47). Comparison of the survival and bacterial load in knockdown beetles with their corresponding controls in each single-, double-, or triple-knockdown experiment confirm this phenomenon. We find that silencing of three components of the AMP cocktail has stronger effects on host survival and bacterial load than knockdown of one or two components, and that the fate of the host-pathogen interaction in our system is determined relatively early in the infection process.

Separate analysis of the single knockdown experiments shows that the effect of *TmAtt1a* knockdown neither increases bacterial load nor lowers host survival while bacterial load in the control increases gradually over three days post-infection. This was supported by the results of the double-knockdown of *TmAtt1a* in combination with *TmAtt2*, which does not seem to affect the efficiency of the latter. By contrast, both knockdowns of *TmAtt1b* and *TmAtt2* dynamically affect bacterial load over time. Indeed, *TmAtt1b* knockdown increases bacterial load at both 1- and 3-days post-infection, while knockdown of *TmAtt2* increases bacterial load only at 1-day post-infection. Interestingly, both these single knockdown treatments result in reduced beetle survival. Silencing *TmAtt2* caused mortality within 15 days which was slower than in *TmAtt1b* knockdown females which died within 10 days (**Figures 2**, **3**). However, overall mortality was similar. These results suggest the importance of the timing of AMP production in controlling of bacterial infection and highlight that the survival differences in knockdown Attacin females can be attributed to differences in their bacterial burden (46).

In the case of the double knockdowns, we observed that ds*TmAtt1a-Att2* beetles showed an initial increased load of *P. entomophila* (1-day post-infection) but of lesser magnitude compared to the single knockdown of *TmAtt2* and resulted in no detectable changes in host survival. In this regard, the increased bacterial burden might be responsible for the early host mortality, whereas a later decrease in bacterial load did not influence mortality. Similarly, double-knockdown of *TmAtt1b-Att2* could be responsible for the drastic increase in *P. entomophila* colonization which on the contrary does not result in an increase in mortality rate. Therefore, both double knockdown treatments increased bacterial loads at later time points after infection but did not influence host survival.

*P. entomophila* infection resulted in the death of the majority of triple-knockdown females within 3 days (**Figure 6A**), leading to a low sample size in the bacterial load experiment at 3-days post-infection (**Figure 6B**), compared to single- and double-knockdown groups. Moreover, depletion of three Attacin gene transcripts led to increased bacterial colonization at 1- and 2-days post-infection. This is earlier in the infection process compared to other knockdown females. We only see an effect of *TmAtt1a* in our triple-knockdown experiments. However, due to the lack of information about the molecular mechanisms of Attacin interactions, we cannot decipher whether this effect comes from a synergistic action of *TmAtt1a* with *TmAtt1b* and/or *TmAtt2* or an efficiency of *TmAtt1a* only when combined with *TmAtt1b* and *TmAtt2*.

It seems that the common denominator between the experiments in which host survival is affected is an increase in bacterial load on the first day after infection. Therefore, only effect size analysis allows for the correction of knockdown treatments by control treatment mortality and provides a meaningful comparison between experiments. Consistent with this, a striking finding of our dataset is that despite the fact that most mortality in these series of experiments occur within five days of infection (**Figures 1**–**6**), the outcome of the host-pathogen interaction appears to be fixed on the first day, as there is a concordance between bacterial load at this time point and mortality.

The relationship between pathogen load and host mortality is known to be affected by pathogen tolerance as well as pathogen resistance. The former has been defined as the tendency of the host to limit the effects of infection to a certain level, resulting in less loss of fitness so that the host lives longer. Resistance, on the other hand, actively reduces the growth of the pathogen (47, 48).The concordance we see indicates that the effects we observed on host survival are likely not due to changes in tolerance to the *P. entomophila* infection, but rather to changes in the ability of beetles to control *P. entomophila* load, or resistance. Our data also show that the number of CFUs, which appears to be critical for mortality is approximately 1 × 10^6^ CFUs per beetle at 1-day post-infection, which is consistent with our preliminary data showing a clear dose-response (**Figure S2**).

This is consistent with the observations of Duneau et al. (46), who suggested that the faster the immune system is activated against infection, the higher the chance of *D. melanogaster* surviving. If immune responses are too late, flies will die when bacterial colonization reaches a certain level (46). Taken together, these findings are consistent with the fact that the risk of mortality increases markedly with increasing bacterial load early in the infection process, rather than just before host death (49). Interestingly, this observation was not made in Zanchi et al. (3), where *T. molitor* was exposed to an opportunistic pathogen. In this case differences in bacterial load were seen much later in the infection process. This co-occurrence between bacterial load and host death has also been observed in *Drosophila* (34, 46).

Following this reasoning, we focused on the effects of the different knockdown treatments on early bacterial load (1-day post-infection) and on host survival in response to *P. entomophila* infection to investigate the kind of interactions that might exist between our three focal genes. First, we note that while *TmAtt1a* alone has no effect on bacterial load and host survival, it is effective in combination with *TmAtt1b* and *TmAtt2* (*TmAtt1a-Att1b-Att2*). Knockdown of *TmAtt1a* in combination with either *TmAtt1b* or *TmAtt2* is inefficient (*TmAtt1a-Att1b* or *TmAtt1a-Att2*). This indicates an antagonistic effect of *TmAtt1a* on *TmAtt1b* and *TmAtt2*, however, the difference between the effect sizes of the knockdown treatments is not significant. Therefore, the datasets of the single- and double-knockdowns do not allow us to decide whether this pattern is the result of antagonism, redundancy, synergy, or an additive effect between the three *TmAttacins* in the context of a double-knockdown.

The effect of triple-knockdown on host survival and *P.entomophila* load is greatest at 1-day post-infection. This observation seems to rule out the possibility of an antagonistic interaction and suggests an additive effect of these three members of the *TmAttacin* family. The effect of the triple-knockdown, although larger, is not significantly different from the effect of the single knockdown of *TmAtt1b*, which could imply that the effects observed with the triple-knockdown treatment could be due exclusively to *TmAtt1b.* However, this seems unlikely because we do not observe a survival phenotype with the double-knockdown of *TmAtt1b-Att2*.

A closer look at the value of the effect sizes on the CFU counts at 1-day post-infection shows that the effect size of the triple-knockdown on the CFU counts is close to the effect size of the three single knockdowns combined (Hedge’s g *TmAtt1a* + *TmAtt1b* + *TmAtt2* = 2.39 *vs*. 2.23 for the triple-knockdown). This is not the case for the double-knockdowns, where, on the contrary, the effect sizes of the double- knockdowns are close to the effect sizes of the corresponding single-knockdowns minus each other (Hedge’s g *TmAtt2* – *TmAtt1a* = 0.47 *vs*. 0.60 for the double-knockdown; *TmAtt1b* – *TmAtt2* = 0.52 *vs*. 0.37 for the double-knockdown). This suggests that the effects of single-, double-, and triple-knockdowns of our three AMPs alter the nature of the interactions between them.

These two possibilities were previously addressed by Hanson et al. (13), who knocked down a larger number of AMP genes across several gene families in *D. melanogaster*. This highlighted additive or synergistic anti-Gram-negative and antifungal activities between AMPs as well as *in vivo* specific AMP-pathogen interactions (3, 13). In a further study Carboni et al., 2021 used the flies from (13) to investigate the role of cecropins in fly immunity. While the knockout of all cecropins did not alter mortality of infected flies, the combined knockdown of the 4 cecropins with 10 other AMPs resulted in significant mortalities, further supporting the idea of higher-order interactions of AMPs (13, 50). Our data confirm that this is also the case in other insect species such as *Tenebrio molitor*. Our data also concur with *in vitro* experiments carried by Yu et al. (12), who reported strong synergistic effects of combinations of three or two AMPs from different families and host organisms, with synergy being stronger for combinations of three than for combination of two AMPs. This was also demonstrated *in vivo* between AMPs of different classes in Zanchi et al. (3), where the burden of an opportunistic pathogen in *T. molitor* is more efficiently controlled by a combination of AMPs than by single ones (3, 12). Overall, our knowledge on AMP interactions *in vivo* is still very limited, as studies have either knocked out groups of AMPs, for example (51) knocked out 10 Bomanins simultaneously, or the groups of AMPs knocked out by Hanson et al., 2019 (13, 51). The approach we presented here and in a previous publication (3), aims to knockdown all combinations of three different AMPs, here within one family. Future work needs to show what the nature of the interactions of AMPs *in vivo* is, as determining synergy for example is conceptually and empirically challenging (52).

To our knowledge, whether the depletion of transcripts of some AMP genes could be compensated by the upregulation of others has never been investigated, which would be an important direction for future research. We did not find evidence for compensation of the depletion of transcripts of *TmAtt1a* and *TmAtt1b* by overexpression of one other of our focal AMPs. Nevertheless, we cannot exclude that other members of the Attacin family or of other AMP families would be upregulated following the KD of one or several *in vivo*. This would add another level of interactions. In spite of this, our work demonstrates, that at least within the Attacin group, when AMPs are knocked down, the overall Attacin response in reduced, rendering beetles more susceptible to infection.

## Data availability statement

The original contributions presented in the study are included in the article/**Supplementary Materials**. Further inquiries can be directed to the corresponding author.

## Author contributions

Conceptualization: MK, CZ and JR. MK designed and performed the experiments. MK and CZ analyzed the data and wrote the manuscript. JR revised the manuscript and procured reagents and materials. All authors contributed to the article and approved the submitted version.
