# Supplementary material for: The effect of combined knockdowns of Attacins on survival and bacterial load in Tenebrio molitor
Source: Front Immunol. 2023 Mar 28;14:1140627. doi: 10.3389/fimmu.2023.1140627 (PMC10090678; doi:10.3389/fimmu.2023.1140627)
Supplement: Supplementary file 1 [file DataSheet_1.docx]

Supplementary Material

The effect of combined knockdowns of Attacins on survival and bacterial load in *Tenebrio molitor*

**Maryam Keshavarz^1†*^, Caroline Zanchi^1†^, Jens Rolff^1^**

*** Correspondence:** Maryam Keshavarz: [mariakshvrz@gmail.com](mailto:mariakshvrz@gmail.com)

# Supplementary Figures and Tables

## Experimental design


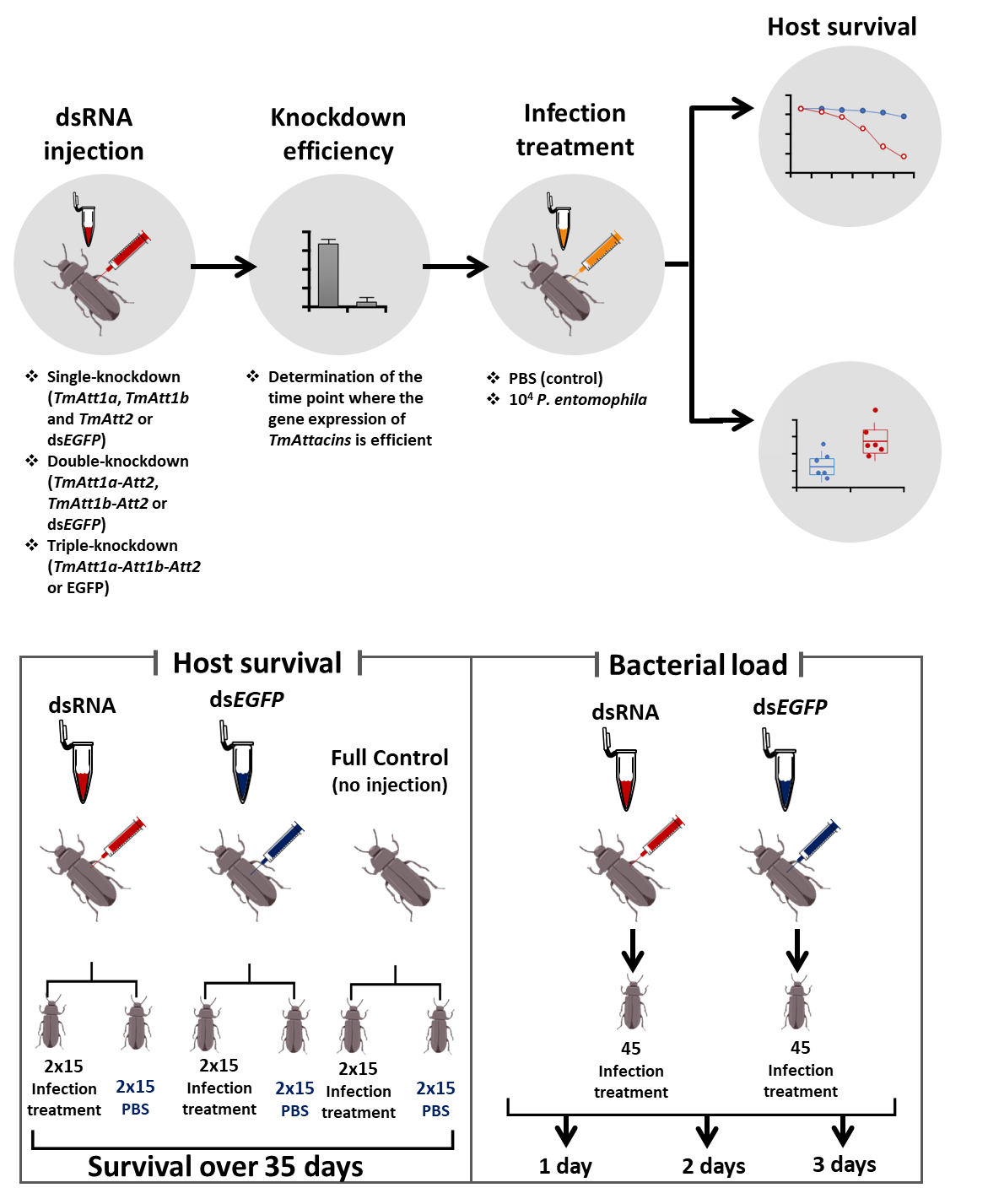


**Supplementary Figure 1.** Schematic illustration of experimental procedure

## *Tenebrio molitor* survival upon *Pseudomonas entomophila* infection


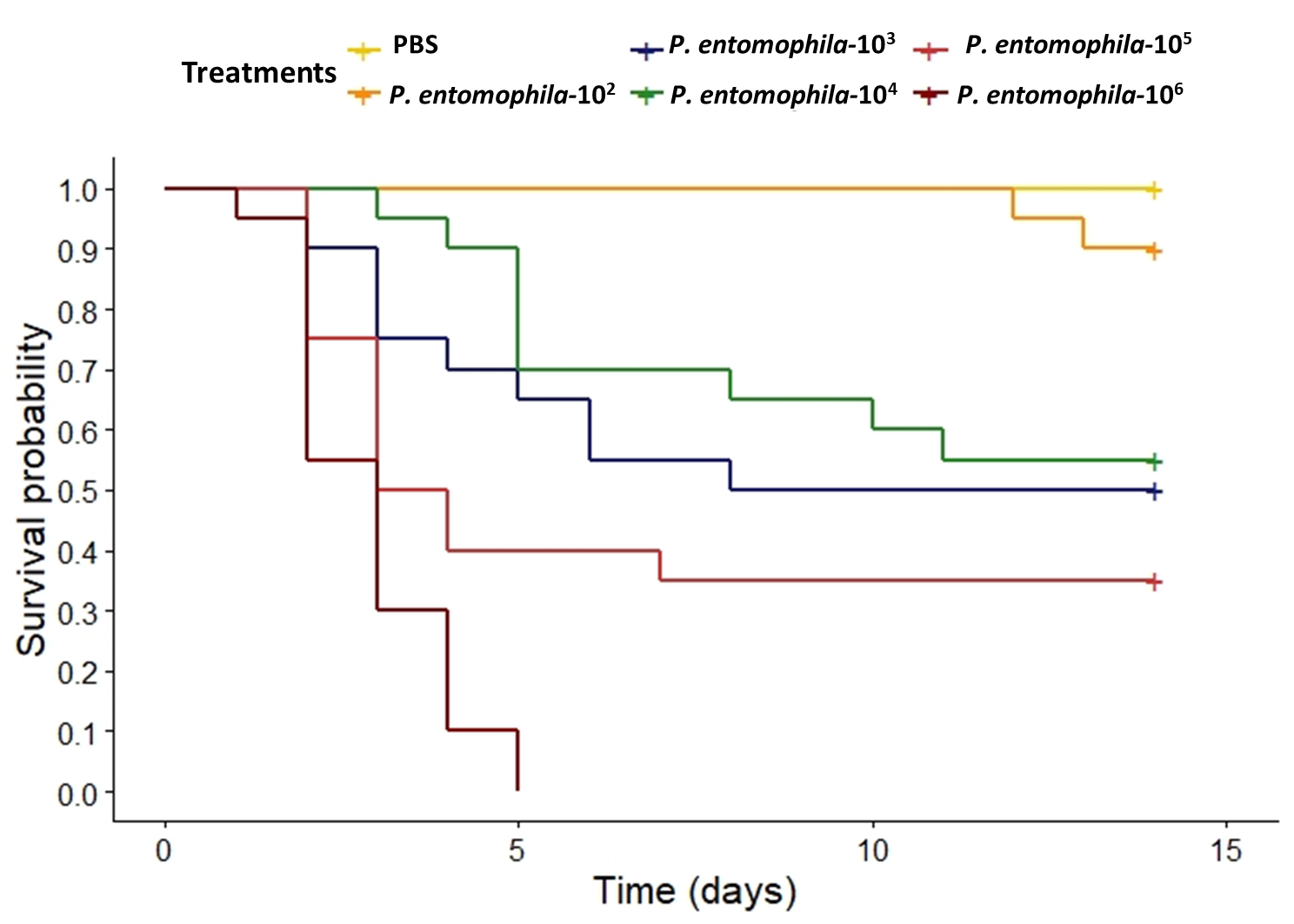


**Supplementary Figure 2.** Survival of *Pseudomonas entomophila*-challenged *Tenebrio molitor* females in 14 days. Different concentrations of *P. entomophila* were injected into adult females (9 to 11-day-old). PBS was injected as a control (n = 10 per treatment group, two biological replicates).

## Sequence of Attacin gene family

**GGTTACCAGGATGCCACCGTTCAAGGGAAAACTTTGGATTGATTGGTACGCAAGGTGTGGGTAGAGTTCATCCATAGAAGACAAACAGAAACAAGTCAAGATGCAGAAACAACTCATTGTCT**

**CAATTCTCGCATTCGCCTCTTTGGCGTTTGCCACGGCGGACAATAAAATTCCTCCTCCCAA**

**>>>>>>>>>> *TmAttacin1a*-qPCR-Fw**

**ACCGGAAGATGGTCAAAGGGAAACGAAATGGAAGGTGGAAGATCCCGGAATCATAAATCTT**

**>>>>>>>>>> *TmAttacin1a*-T7-Fw**

**CAGCACCGCGAGAAGCTTTATGAAAGTGGTCCCCACCGATTCGACGCCACTGCTGCTTATAAGAAGAACTTTGTTGACAAAATGGATCCTGCCAGGACCATTGCGAGAGTCGACTACAAATA**

**<<<<<<<<<*TmAttacin1a*-T7-Rv**

**CCTTCCAGGAGATACTAGTCTGGGTGTACAAGCCGAAAACATTCAGCGCTTCGGTACTGTA**

**<<<<<<<<<<*TmAttacin1a*-qPCR-Rv**

**TTGTCTGCCGAAGCAACACGTAATCTCTACAAGGATCGAAAGAGTTCTTTGGATGTTGGTGTCAACTACGGACAAACTTTTTCACCGTTTGTCAGAAGCGAACCTTTCTTTGGAGGATTCGTCAGAGGAAGATTCTAATTTATTAGTGAAATAGAATTTTGCCAAGCTCTTGCCGTAGACAATTGCTCAATTTTAAATTAATTTACTTAATTGTTCTTACGTTTGTTTATTAATTAATTTATTAATTCAAATACAATAATTATTTTTATCTATGTTTTCTATATTTGGAATAAAAGTAAAAACCCC**

**Supplementary Figure 3.** The complete cDNA sequence of *TmAttacin1a*. The position of all primers used for real-time analysis (blue) and dsRNA synthesis (red) are marked. Final 220-bp PCR product containing the T7 promoter sequence was synthesized.

**AAAACGAATAACTAATCAATGAACATGCAAACAGTGTATATCATTGCATTGTGCTGTTTGGCATCAGCTT**

**TAGCCAGACCCGGCAACACCAAGCCAGAAGACCAAAGTCAAACAAAATGGGGAGTACGAGATGGAGTTTT**

**>>>>>>>>>> *TmAttacin1b*-T7-Fw**

**AAATGTTGAGCACCACGGAAATCTTTACAAGAATGACAATCATCGATTCGACGGTACTGCCTCCGTTACT AAGAATTTTGTCGACAACAAAGATCCTCTCTTAGTGGGAGGTCGCGTAGATTATAAACATCTCCCTTCGA**

**>>>>>>>>>> *TmAttacin1b*-qPCR-Fw**

**ATTCTGCCATCGGTCTGGGAGCTGTGAATGCAGGACAATTTGGTACTAAAGTGGATCTGGAAGCATCGCG**

**<<<<<<<<<<*TmAttacin1b*-T7-Rv**

**TACCCTCTTCAAAGACAGGTTCAGTCAGTTTGATGCTGGAGTTAGCTACGGACAGCGATTCGGGGGACCG**

**<<<<<<<<<<*TmAttacin1b*-qPCR-Rv**

**TTTGGCAACAGCGAACCGGTGTTTGGAGGTTTCATCAGAGGGAGATTTTAATTTTTAATTTGTCAACAAT TTGTAAATTAATAAAAGCGTAATTTATTTGTATCTCATGATTTATTTATTGTAACATTAGCAAGAATAAT TATATCAAATATTCTTTTGTTACAATGCAAATTCCTACCCATAGATTCTGACTGTGATATTTTTTAAAGG CGGAAGTACTGAGCGATCAAGAAGTAATATTAGATTTGTTTCTTAAAAGATATTATAATTTCAATAAAAG AACAGCACGGAATTTAGGCCGAATTTATTTATACAATATTTATTAAATAGTGATACATAATTTCTTTGAA AGTACTATTTTTTTAATATTTTTTATTTATTTTAGTATGCGCCCACATTTTACACGACATTTACGATTCA CGTCAGAATTTGTTCATACGAATAATACAGAGGTGATTACTGATTATACATGAAAATTAAATTTGTATAC ATTATTTGTTTGTTAAAACAAAAATAAATGTAAAATACAAAGTTCACTTTTTGCTGCAACTTTTATCTGT TTGGCTAAT**

**Supplementary Figure 4.** The complete cDNA sequence of *TmAttacin1b*. The position of all primers used for real-time analysis (blue) and dsRNA synthesis (red) are marked. Final 205-bp PCR product containing the T7 promoter sequence was synthesized.

**GTTAACCGTAAATTCATTCTTTTGCAAAAACCACAGATCAGAAAGAAATGGCAAAATATTTTTTCTAACA TCATTTAGCAATCGCGATGTTTTAAATCTGCAATTGGTTAATCATTTATTAAACGTTGTTTGTTGCACTG AATTCATTTTCGTTTTATCCAGTTTTATACATCTCCTGAAAAATTACATTTACTCGTCTTACATTGTTTC**

**>>>>>>>>>> *TmAttacin2*-T7-Fw**

**AACAACCATCTCTGCAAAAATGTTCAAGCTCATCGTCCTCGCTCTTGTCGGCCTCGCTGCAGTGTCAGCC**

**TACGAAGTGGTCCAAGATGACCAAGGTCAAGAATTCTTCTTGGTGCCTCTACACCGGCAACGAAGACAGA CCAGTGTGGACATCTCCAAGTCGAATCCTGGAGTTAGGGCGACGGTCAGCCATCAAGGTACCATCTTCAA**

**<<<<<<<<<<*TmAttacin2*-T7-Rv**

**CAACGGAGACCATCGTCTGGACGGTGGGGCCTTCGCTTCGAAGCAGTTCCGTCCATCTGGACCCGCCACG**

**>>>>>>>>>> *TmAttacin2*-qPCR-Fw**

**GTTGGGGGGAAACTGGGATATTCGCACGTCCCTTCGGGTTCCGGTTTGAACGTGGGGGCCCAACGCACTC AACGGTTCGGTACGGACGTCAGCGCCACGGGAAATGCGAACCTGTGGAGGAGGGGCAACGCCCGTCTGGA**

**<<<<<<<<<<*TmAttacin2*-qPCR-Rv**

**TGCTGTTGGCCAGTACAACAGACATTTCGGAGGGGTCGGCGGGACCGGAAGGCCCAACTATTACGGAGGT CTCCAGTTCTCCCACAGATTTTAATTTATTTATTTATTTCAATTCCTACTTAATCTATAATCATTAAATC CAATTGCTCCACTACTGCCTCCTCCGATTCCTCCACCACTGCCTCCTCCAAGGCCGCTGCCTCCTC**

**Supplementary Figure 5.** The complete cDNA sequence of *TmAttacin2*. The position of all primers used for real-time analysis (blue) and dsRNA synthesis (red) are marked. Final 254-bp PCR product containing the T7 promoter sequence was synthesized.

## Expression of *TmAttacin* genes upon *Pseudomonas entomophila* infection


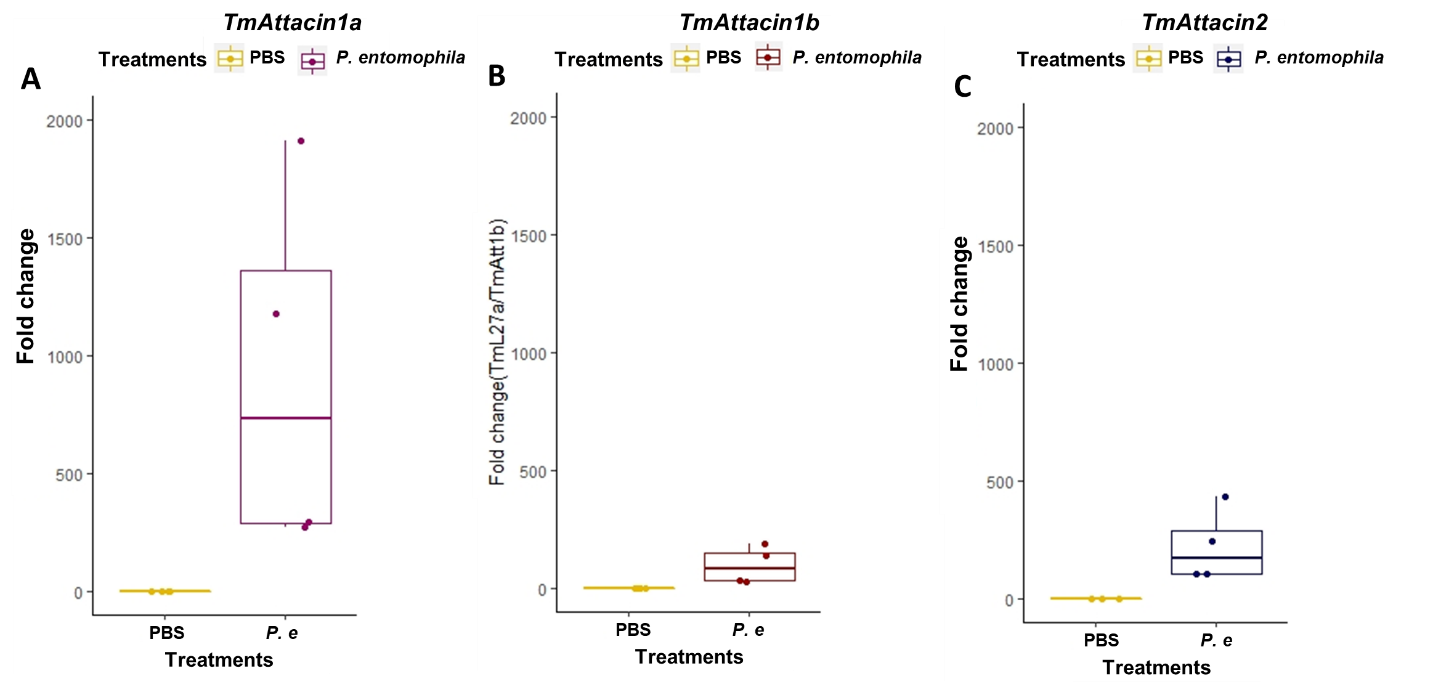


**Supplementary Figure 6.** Relative expression profiles of *TmAttacin1a* (**A**), *TmAttacin1b* (**B**), and *TmAttacin2* (**C**) in the whole body of *Tenebrio molitor* females after challenging with *Pseudomonas entomophila* (*P. e*) (n =4 per treatment group, two biological replicates) at 24 h post-infection was examined by qRT-PCR. PBS injected groups used as mock controls. *T. molitor* 60S ribosomal protein L27a (*TmRPL27a*) was used as an internal control. In the box plots, the lower (first) quartile is the closest boundary to zero, the line within the box marks the median (second quartile), and the upper (third) quartile. Dots indicate the data. The Ct values of the gene of interest were normalized to the Ct values of the reference gene.

## Knockdown efficiency of RNAi-treated females *Tenebrio molitor*


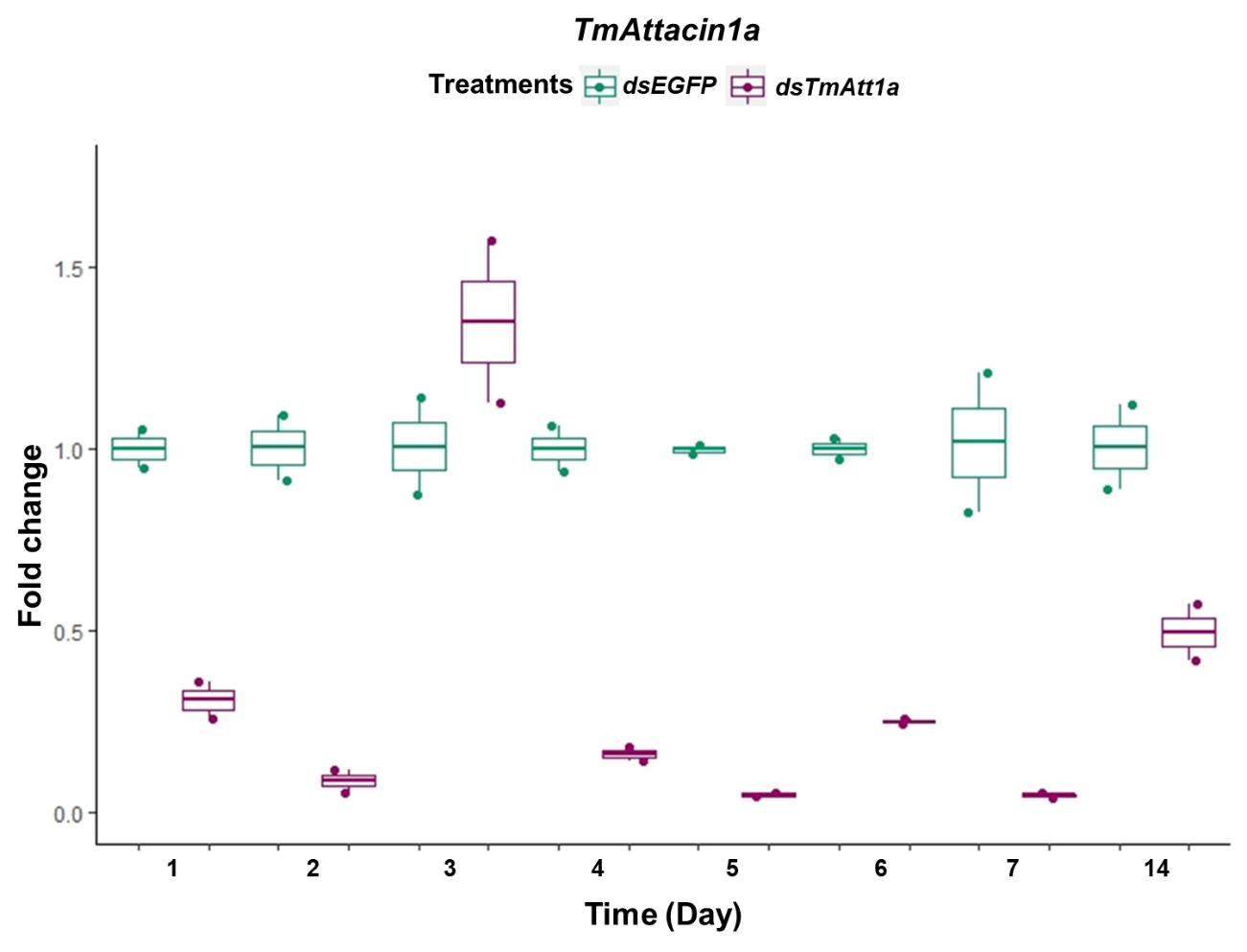


**Supplementary Figure 7.** Quantitative measurement of *TmAttacin1a* (*TmAtt1a*) mRNA level in ds*TmAtt1a*-injected *Tenebrio molitor* adult females determined by RT-qPCR. One microgram of dsRNA targeting *TmAtt1a* at the concentration of 500ng/μL in 2 μL were injected to females (9 to 11-day-old) *Tenebrio*. Total RNA was isolated on the 1^st^, 2^nd^, 3^rd^, 4^th^, 5^th^, 6^th^, 7^th^, and 14^th^ day after treatments (n = 4 pools of four beetles each per treatment group per time point). The mRNA quantity of *TmAtt1a* was measured in relation to *TmRPL27a* as an internal control by RT-qPCR. *EGFP* RNAi was used as a negative control. In the box plots, the lower (first) quartile is the closest boundary to zero, the line within the box marks the median (second quartile), and the upper (third) quartile. Dots indicate the data. The Ct values of the gene of interest were normalized to the Ct values of the reference gene.


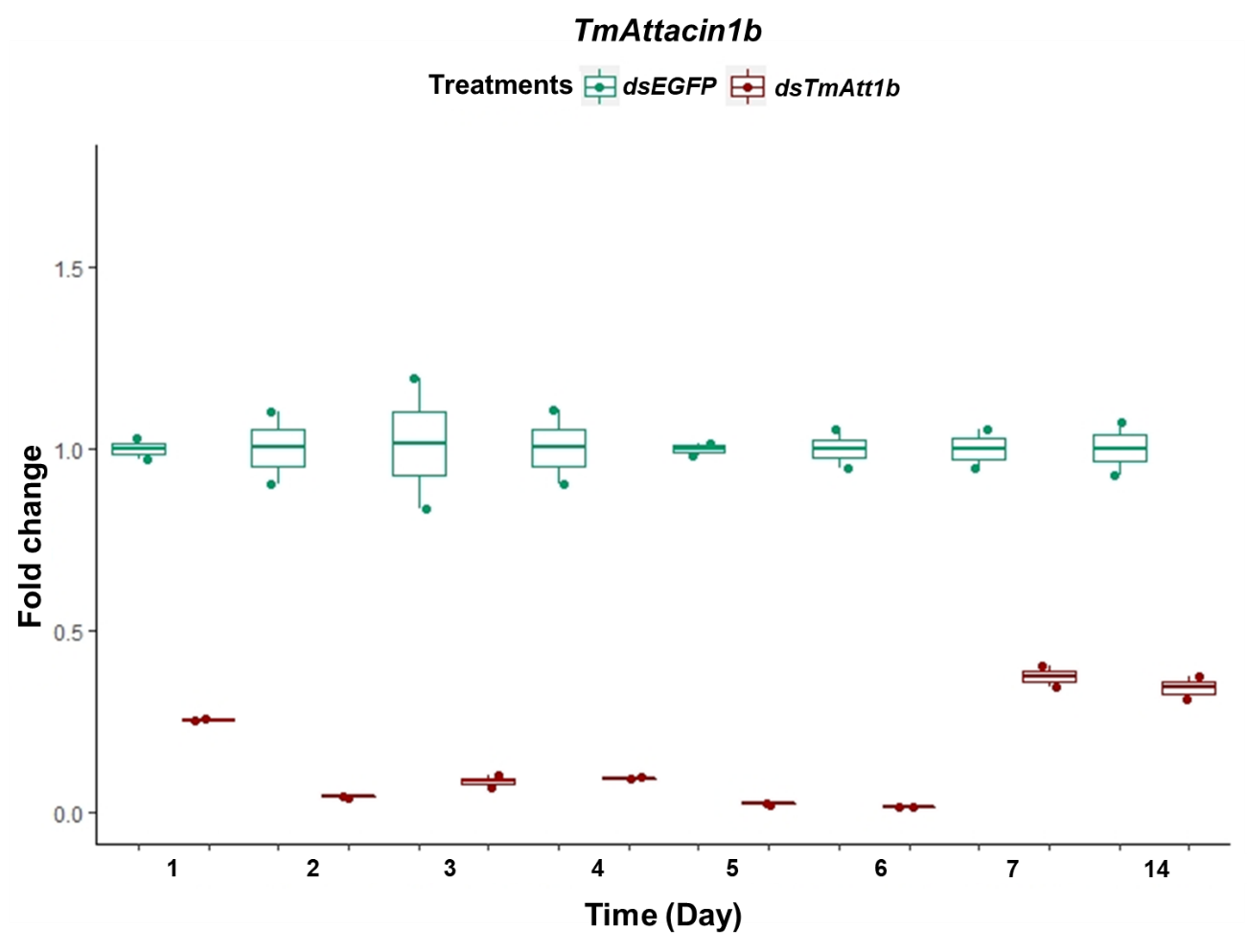


**Supplementary Figure 8.** Knockdown efficiency of *TmAttacin1b* (*TmAtt1b*) in ds*TmAtt1b*-injected adult females on the 1^st^, 2^nd^, 3^rd^, 4^th^, 5^th^, 6^th^, 7^th^, and 14^th^ day post-injection (n = 4 per treatment group per time point) were measured by RT-qPCR. Double-stranded RNA specific to *TmAtt1b* was injected (1 μg) into females (9 to 11-day-old) and ds*EGFP*-injected group served as a negative control. The mRNA transcript of *TmAtt1b* is presented relative to *TmRPL27a* as an internal control. The other details were the same as in Figure S7.


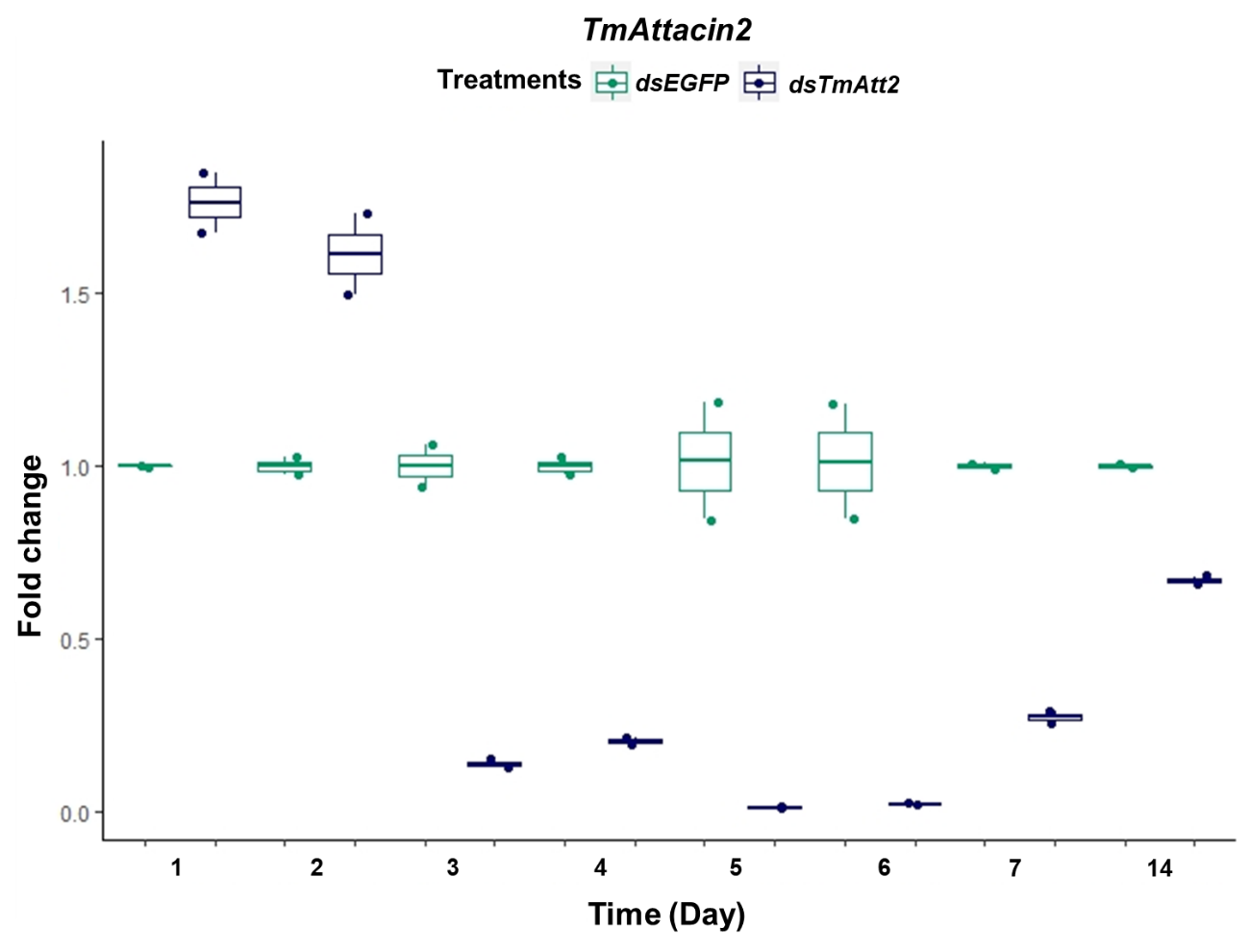


**Supplementary Figure 9.** Double-stranded RNA-induced expression of *TmAttacin2* (*TmAtt2*) in ds*TmAtt2*-injected *Tenebrio molitor* females determined by RT-qPCR. Adult females were treated with 2 μL (1 μg) of either interest gene or control-dsRNA (ds*EGFP*). Total RNA (n = 4 per treatment group per time point) was isolated at indicated time periods (Day-1, -2, -3, -4, -5, -6, -7, and -14). The mRNA values of *TmAtt2* are normalized relative to *TmRPL27a* as an internal control. The other details were the same as in Figure S7.


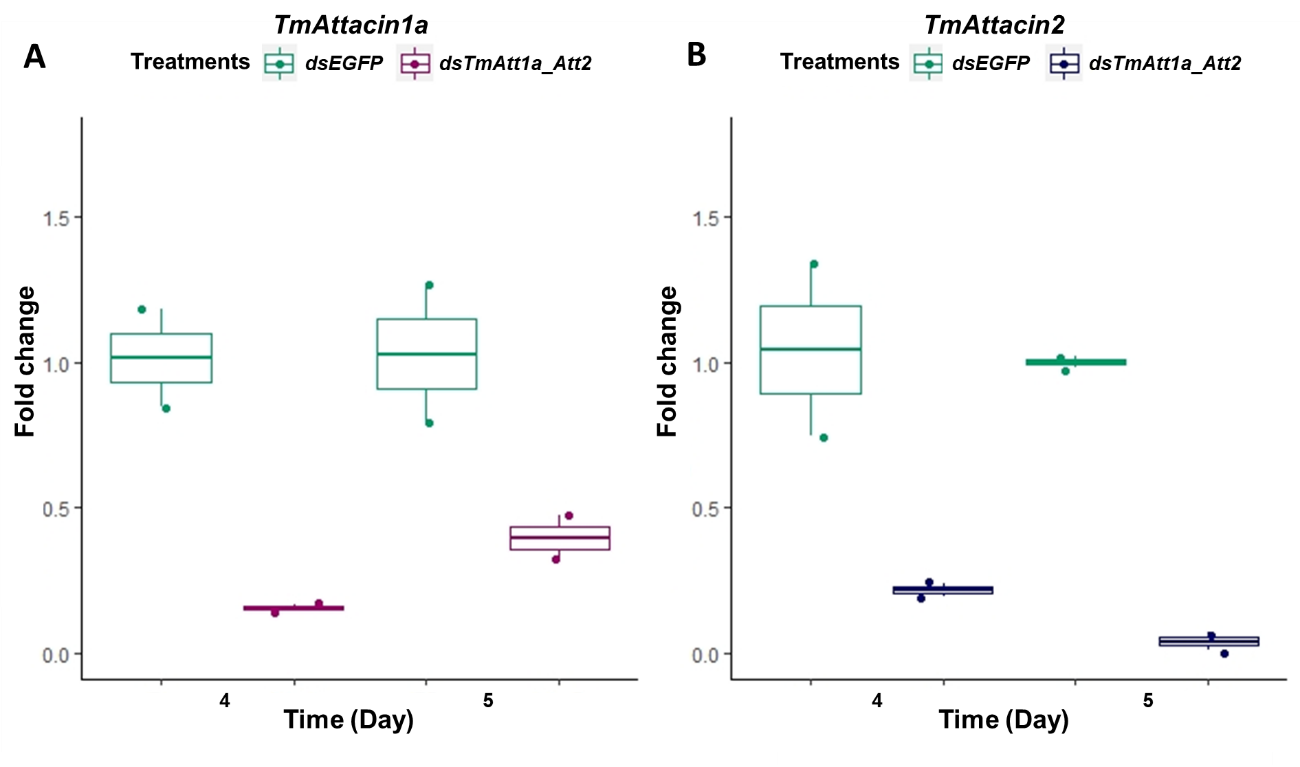


**Supplementary Figure 10.** The RT-qPCR expression of target *Tenebrio molitor* genes was assessed post-RNAi knockdown. The box plots represent the comparison between the fold change values of EGFP (negative control), (**A**) *TmAttacin1a*, and (**B**) ds*TmAttacin2* when adult females (n = 4 per treatment group per time point) were treated with 2μg of ds*TmAtt1a-Att2* at the concentration of 1000ng/μL for each target genes on the 4^th^ and 5^th^ day post-injection. The mRNA transcripts of the respective *TmAttacin* genes are presented relative to those for *TmRPL27a* as an internal control. Data are presented as box depicting median, 25^th^ (first quartile) and 75^th^ (third quartile) percentiles. The Ct values of the gene of interest were normalized to the Ct values of the reference gene. The other details were the same as in Figure S7.


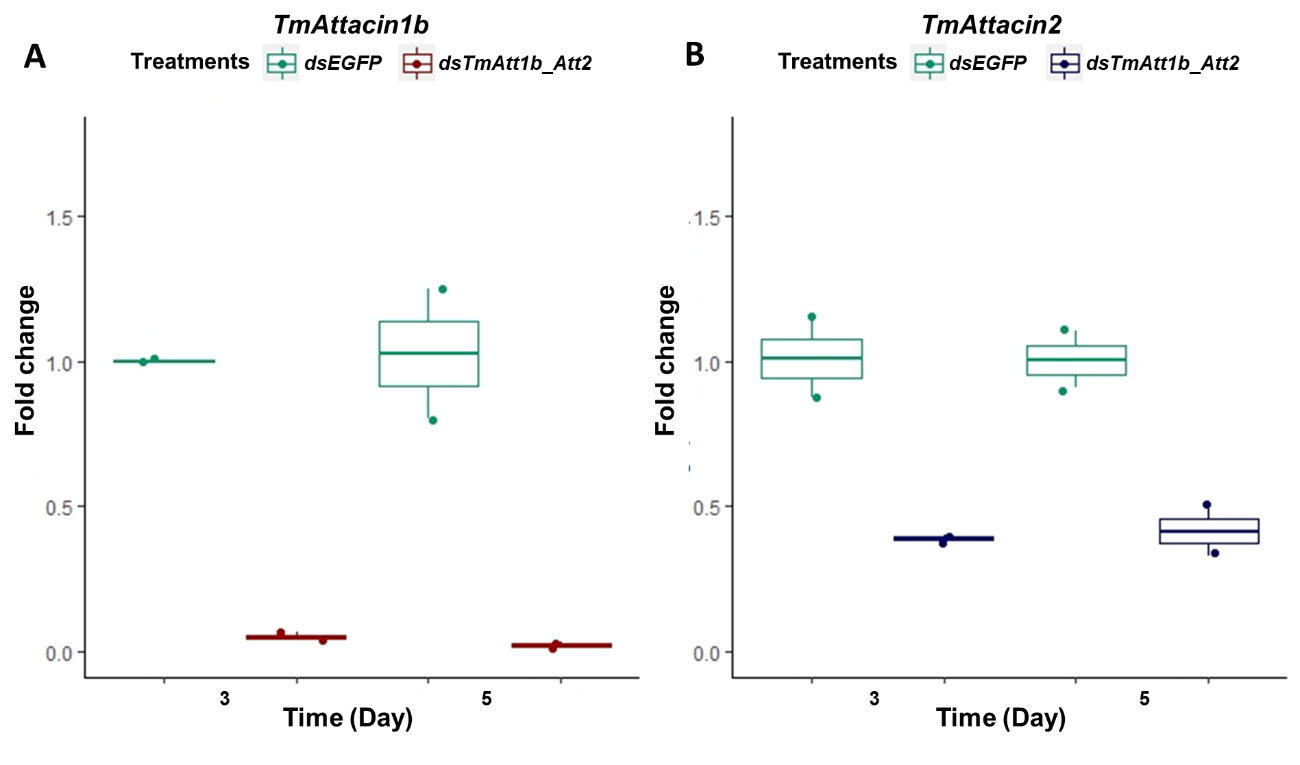


**Supplementary Figure 11.** The silencing efficiency of double RNAi knockdown of *TmAtt1b-Att2* on the 3^rd^ and 5^th^ day post-injection evaluated by RT-qPCR. (**A**) *TmAttacin1b* and (**B**) *TmAttacin2* efficiently reduction-fold change in ds*EGFP*- and ds*TmAtt1b-Att2*-injected females with 2μg of ds*TmAtt1b-Att2* at the concentration of 1000ng/μL for each representative genes determined by RT-qPCR. The mRNA transcripts of the interest genes were normalized using *TmRPL27a* as reference gene. *EGFP* RNAi was used as a negative control. The other details were the same as in Figure S7.


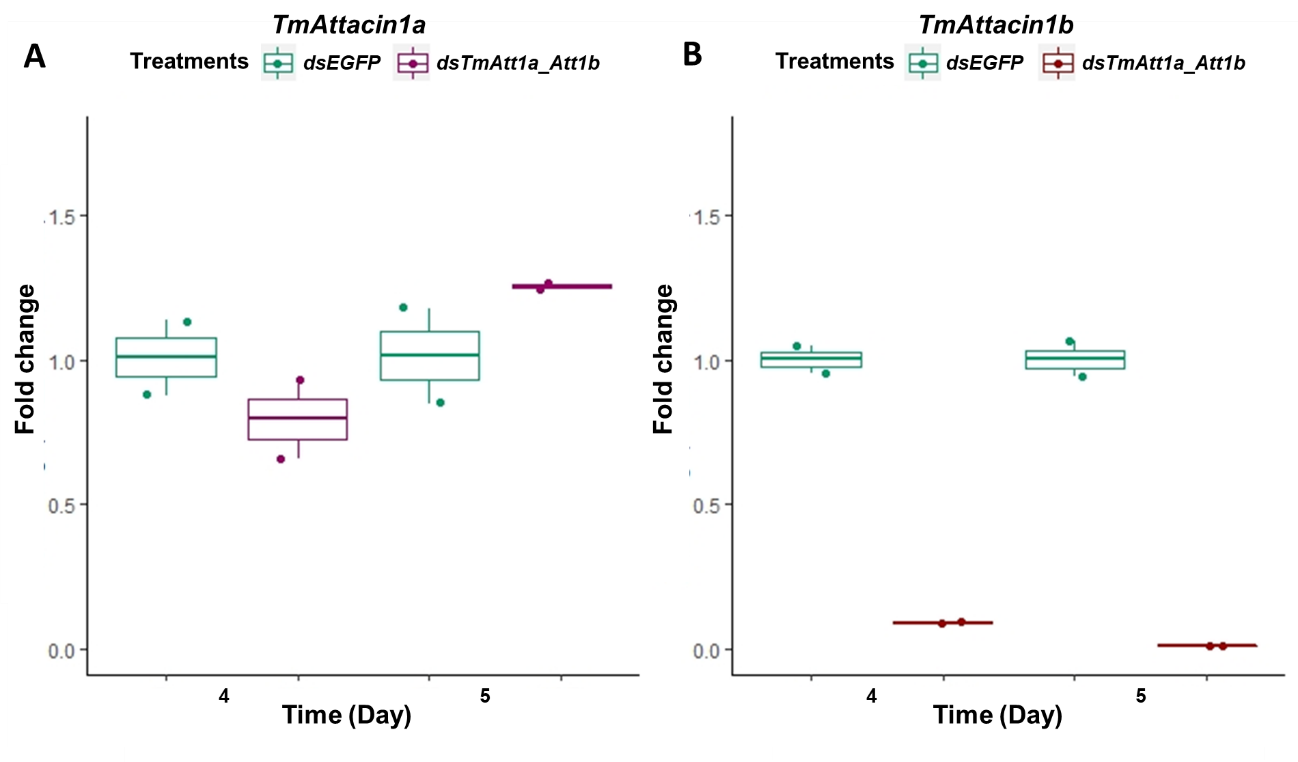


**Supplementary Figure 12.** The knockdown efficiency of double RNAi knockdown of *TmAtt1a-Att1b* on the 4^th^ and 5^th^ day post-injection evaluated by RT-qPCR. (**A**) *TmAttacin1a* and (**B**) *TmAttacin1b* expression level in ds*EGFP*- and ds*TmAtt1a-Att1b*-injected females with 2μg of ds*TmAtt1a-Att1b* at the concentration of 1000ng/μL for each representative genes determined by RT-qPCR. The mRNA transcripts of the interest genes were normalized using *TmRPL27a* as reference gene. *EGFP* RNAi was used as a negative control. The other details were the same as in Figure S7.


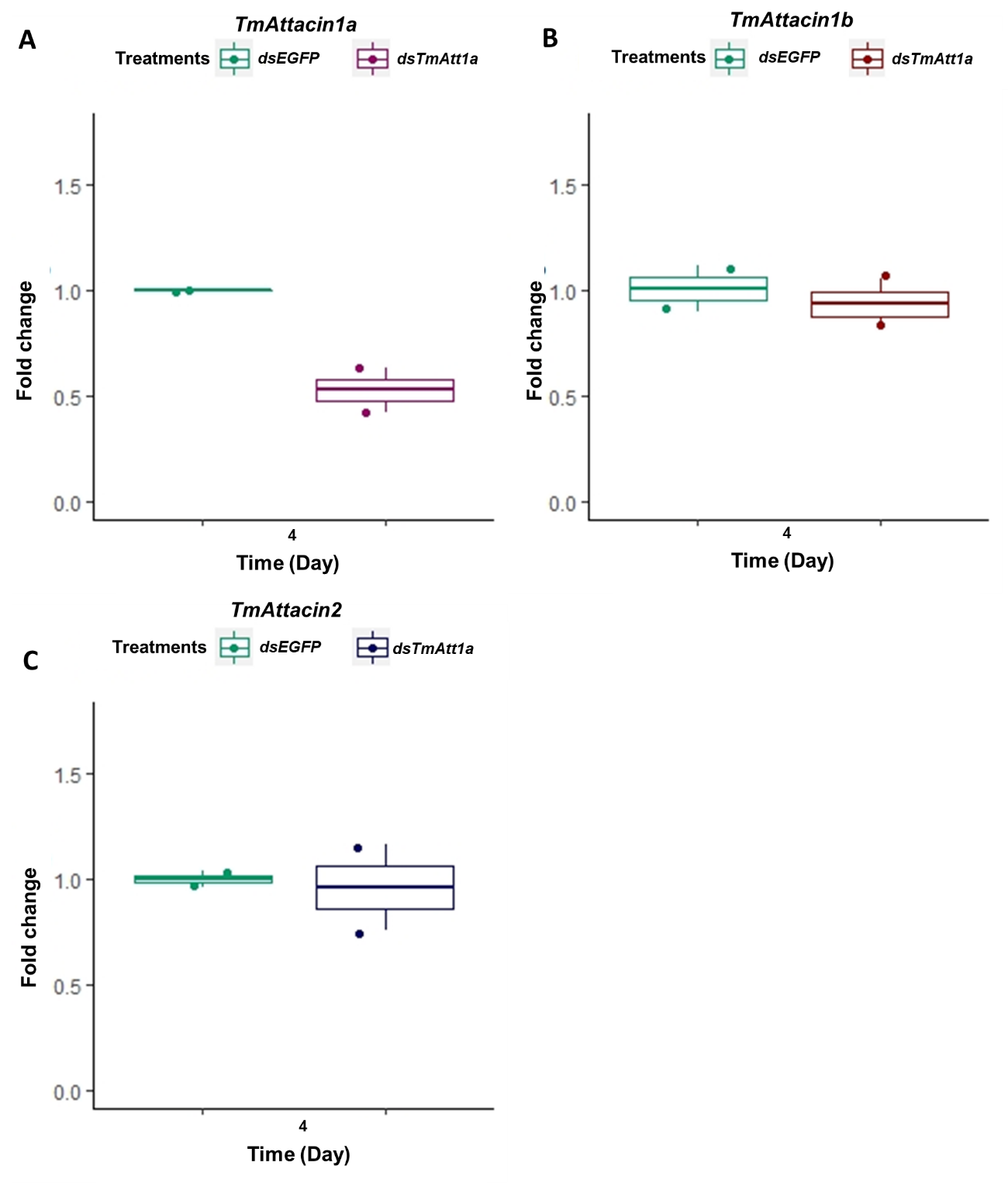


**Supplementary Figure 13.** Relative expression profiles of (**A**) *TmAttacin1a*, (**B**) *TmAttacin1b*, and (**C**) *TmAttacin2* in ds*TmAtt1a*-treated females (n =4 pools of four beetles each per treatment) on 4^th^ day post-injection was examined by qRT-PCR. Females were injected with 2μg of dsRNA at the concentration of 1000ng/μL for both *TmAtt1a* and control. The mRNA transcripts of the interest genes were normalized using *TmRPL27a* as reference gene. *EGFP* RNAi was used as a negative control. The other details were the same as in Figure S7.

**
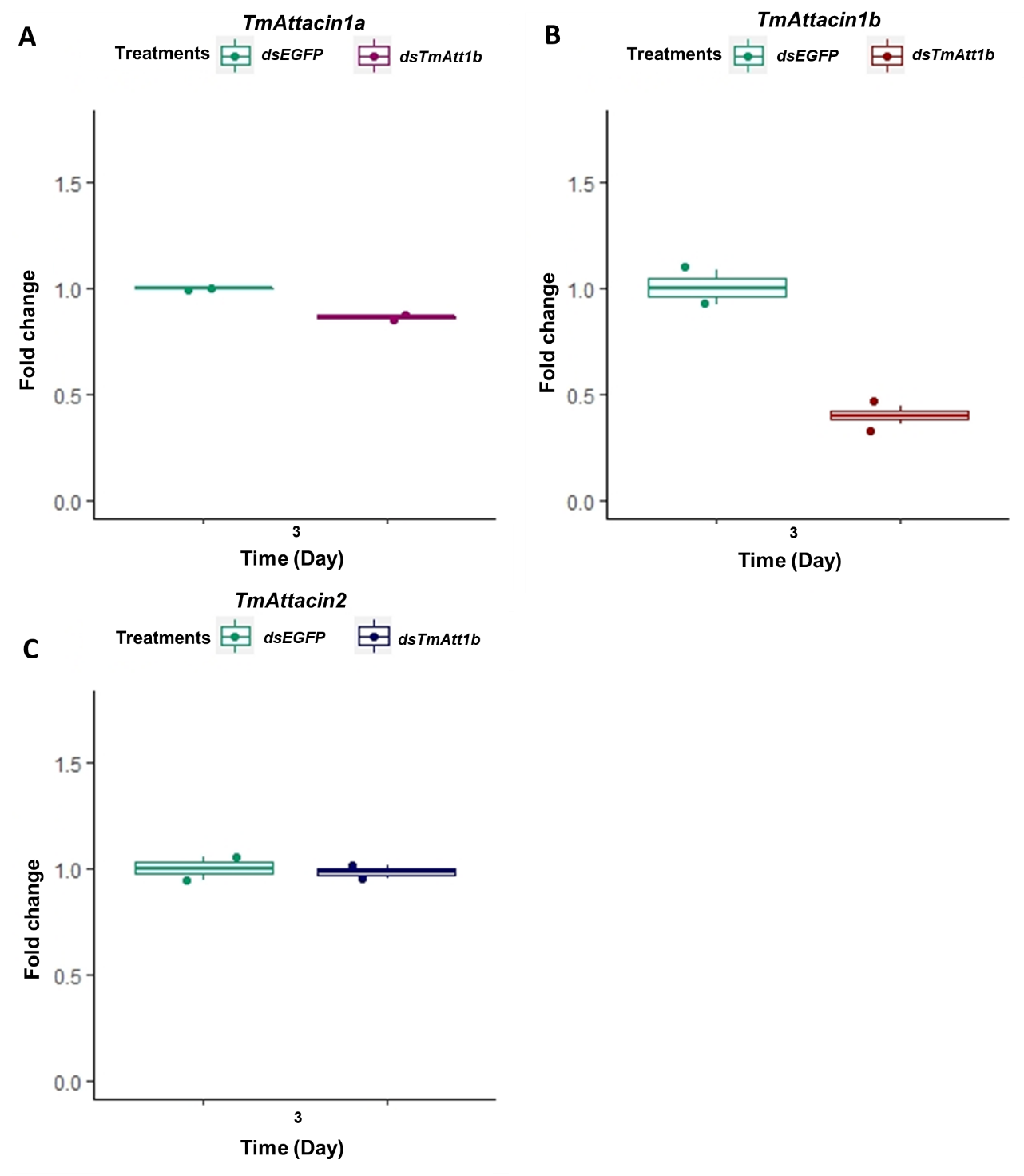
**

**Supplementary Figure 14.** Relative expression profiles of (**A**) *TmAttacin1a*, (**B**) *TmAttacin1b*, and (**C**) *TmAttacin2* in ds*TmAtt1b*-treated females (n =4 pools of four beetles each per treatment) on 3^rd^ day post-injection was examined by qRT-PCR. Females were injected with 2μg of dsRNA at the concentration of 1000ng/μL for both *TmAtt1b* and control. The mRNA transcripts of the interest genes were normalized using *TmRPL27a* as reference gene. *EGFP* RNAi was used as a negative control. The other details were the same as in Figure S7.


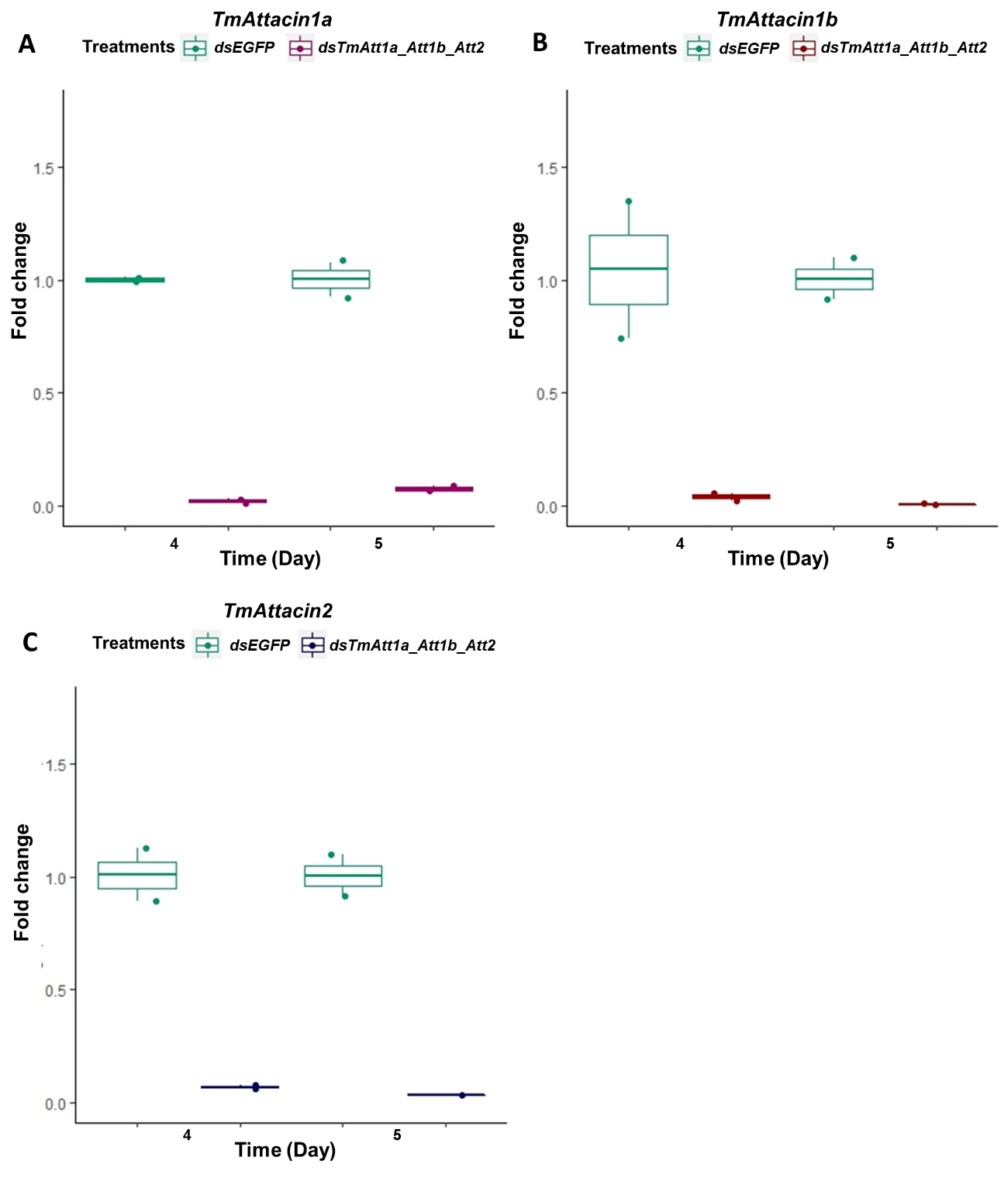


**Supplementary Figure 15.** The fold change of (**A**) *TmAttacin1a*, (**B**) *TmAttacin1b*, and (**C**) *TmAttacin2* mRNA transcripts in ds*TmAtt1a-Att1b-Att2* adult females on the 4^th^ and 5^th^ day post-injection (n = 4 per treatment group per time point) were measured by RT-qPCR. The box plots depict remarkable downregulation gene expressions of *TmAtt1a*, *TmAtt1b*, and *TmAtt2* when adult females were treated with 6μg of ds*TmAtt1a-Att1b-Att2* at the concentration of 1000ng/μL for each target genes compared to ds*EGFP* (negative control). The mRNA transcripts of the respective *TmAttacin* genes are presented relative to those for *TmRPL27a* as an internal control. The other details were the same as in Figure S7.

**
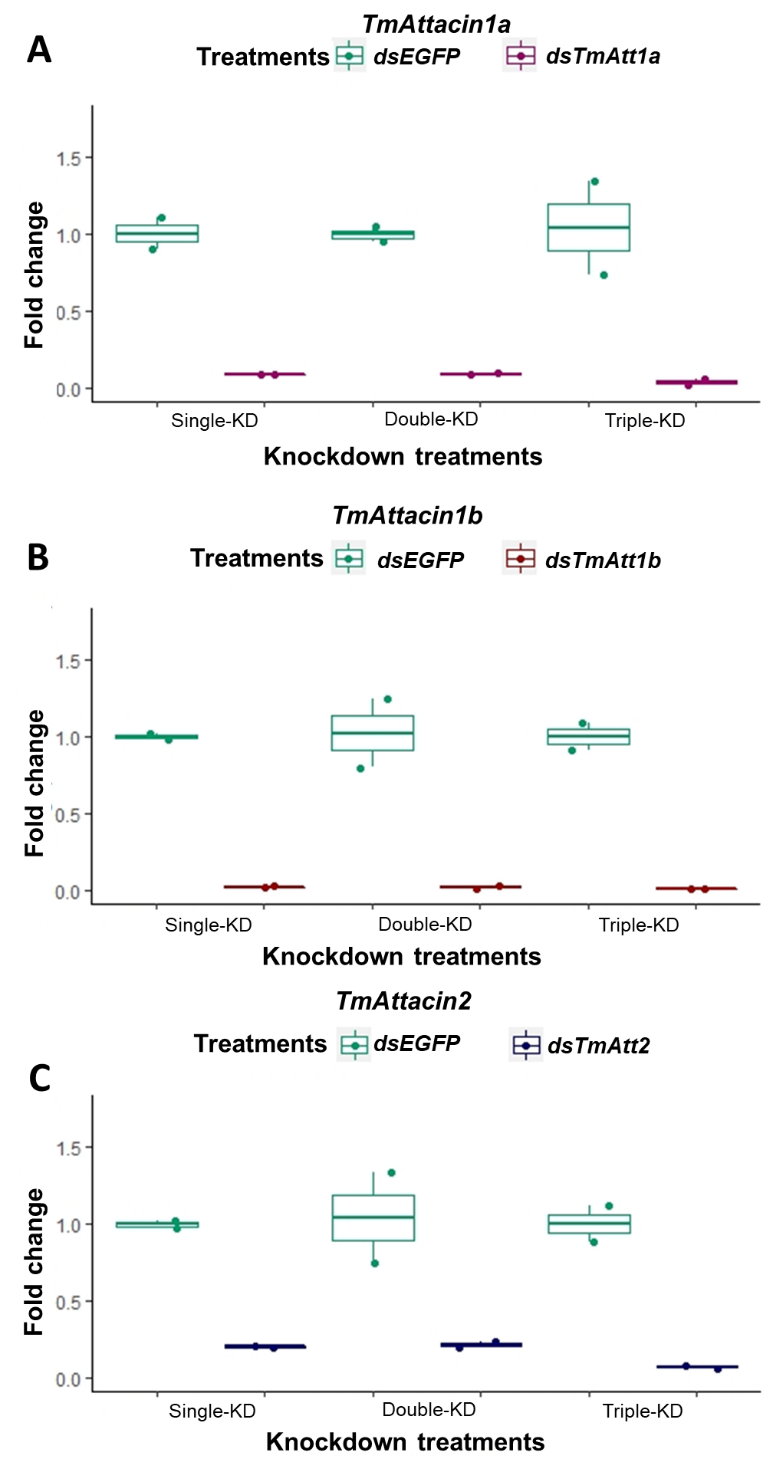
**

**Supplementary Figure 16.** Overview of the fold change of (**A**) *TmAttacin1a*, (**B**) *TmAttacin1b*, and (**C**) *TmAttacin2* mRNA transcripts in single-, double-, and triple-knockdown females on the 4^th^ day post-exposure.


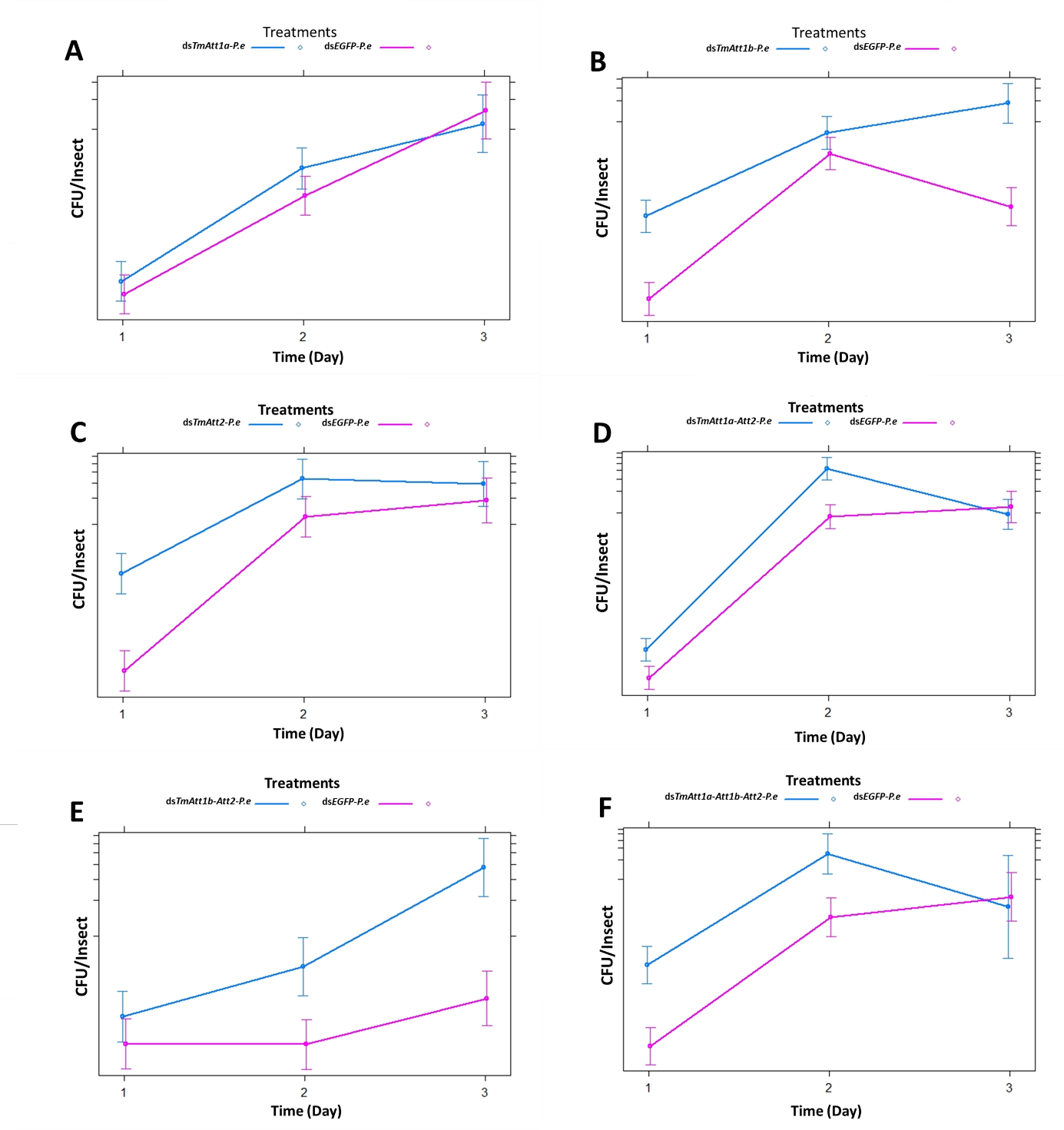


**Supplementary Figure 17.** Effect plots of the predicted probabilities conducted on generalized linear models obtained from CFU counts of *Pseudomonas entomophila* over 3 days. Here, dots and error bars are presented on different treatments including (**A**) ds*TmAttacin1a* females infected with *P. entomophila* (ds*TmAtt1a*-*P. e*), (**B**) ds*TmAttacin1b* females infected with *P. entomophila* (ds*TmAtt1b*-*P. e*), (**C**) ds*TmAttacin2* females infected with *P. entomophila* (ds*TmAtt2*-*P. e*), (**D**) ds*TmAtt1a*-*Att2* females infected with *P. entomophila* (ds*TmAtt1a-Att2*-*P. e*), (**E**) ds*TmAtt1b*-*Att2* females infected with *P. entomophila* (ds*TmAtt1b-Att2*-*P. e*), (**F**) ds*TmAtt1a-Att1b*-*Att2* females infected with *P. entomophila* (ds*TmAtt1a-Att1b-Att2*-*P. e*) and controls (ds*EGFP*). The dots and error bars represent the probabilities, and 95% confidence intervals (95%-CI), respectively. If 95%-CIs overlap by no more than about half of the arm length, there is significant difference between treatments.

## Supplementary Tables

**Supplementary Table 1.** Primer sequences used in this study

| **AMP gene** | **Purpose** | **Forward primer sequence** | **Reverse primer sequence** |
| --- | --- | --- | --- |
| ***TmAtt1a*** | qPCR | 5′-GAAACGAAATGGAAGGTGGA-3′ | 5′-TGCTTCGGCAGACAATACAG-3′ |
| ***TmAtt1b*** | qPCR | 5′-GAGCTGTGAATGCAGGACAA-3′ | 5′-CCCTCTGATGAAACCTCCAA-3′ |
| ***TmAtt2*** | qPCR | 5′-AACTGGGATATTCGCACGTC-3′ | 5′-CCCTCCGAAATGTCTGTTGT-3’ |
| ***TmRPL27a*** | qPCR  (reference gene) | 5′-TCATCCTGAAGGCAAAGCTCCAGT-3′ | 5′-AGGTTGGTTAGGCAGGCACCTTTA-3′ |
| ***TmAtt1a*** | ds-RNA  construct | 5′-TAATACGACTCACTATAGGG GCACCGCGAGAAGCTTTATG-3′ | 5′-TAATACGACTCACTATAGGG  AGTACCGAAGCGCTGAATGT-3′ |
| ***TmAtt1b*** | dsRNA  construct | 5′-TAATACGACTCACTATAGGG GACGGTACTGCCTCCGTTAC-3′ | 5′-TAATACGACTCACTATAGGG TACGCGATGCTTCCAGATCC-3′ |
| ***TmAtt2*** | dsRNA  construct | 5′-TAATACGACTCACTATAGGG  TACGCGATGCTTCCAGATCC-3′ | 5′-TAATACGACTCACTATAGGG CCAGATGGACGGAACTGCTT-3′ |
| **EGFP** | dsRNA  construct (control  dsRNA) | 5′-TAATACGACTCACTATAGGG CTTAATGCACCACCACCACCAC-3′ | 5′-TAATACGACTCACTATAGGG  GTGACCCAGGATGTTACCGTC-3′ |

**※Underline indicates T7 promoter sequences.**

| **knockdown experiment** | ***P. entomophila* infection**  **(Day after  dsRNA treatment)** | **Colony-forming units** | | | | | | **Survival** | |
| --- | --- | --- | --- | --- | --- | --- | --- | --- | --- |
|  |  | **Day1** | | **Day2** | | **Day3** | | **Replicate 1** | **Replicate 2** |
|  |  | ds*EGFP* | ds*Att* | ds*EGFP* | ds*Att* | ds*EGFP* | ds*Att* |  |  |
| ***TmAtt1a*** | 4 | 15/15 | 15/15 | 15/15 | 15/15 | **7**/15 | **7**/16 | 15 | 15 |
| ***TmAtt1b*** | 3 | 15/15 | 15/15 | 15/15 | 15/15 | **11**/15 | **10**/20 | 15 | 15 |
| ***TmAtt2*** | 3 | 15/15 | 15/15 | 15/15 | 15/15 | **12**/15 | **12**/15 | 15 | 15 |
| ***TmAtt1a-Att2*** | 4 | 15/15 | 15/15 | **14**/18 | 15/19 | **8**/15 | **9**/15 | 15 | 15 |
| ***TmAtt1b-Att2*** | 3 | 15/15 | 15/15 | 15/15 | **11**/15 | **13**/15 | **11**/16 | 15 | 15 |
| ***TmAtt1a-Att1b-Att2*** | 4 | 15/15 | 15/15 | **14**/15 | **13**/15 | **9**/15 | **2**/15 | 15 | 15 |

**※Number of dissected females /dsRNA-treated females upon infection (n/n).**

**Supplementary Table 2.** Sample size in single-, double-, and triple-knockdown injected females used to address host survival and the bacterial loads
